# Supplementary material for: A pair of congenic mice for imaging of transplants by positron emission tomography using anti-transferrin receptor nanobodies
Source: eLife. 2025 Aug 18;14:RP104302. doi: 10.7554/eLife.104302 (PMC12360783; doi:10.7554/eLife.104302)

Source Gel, Figure 1A, left panel

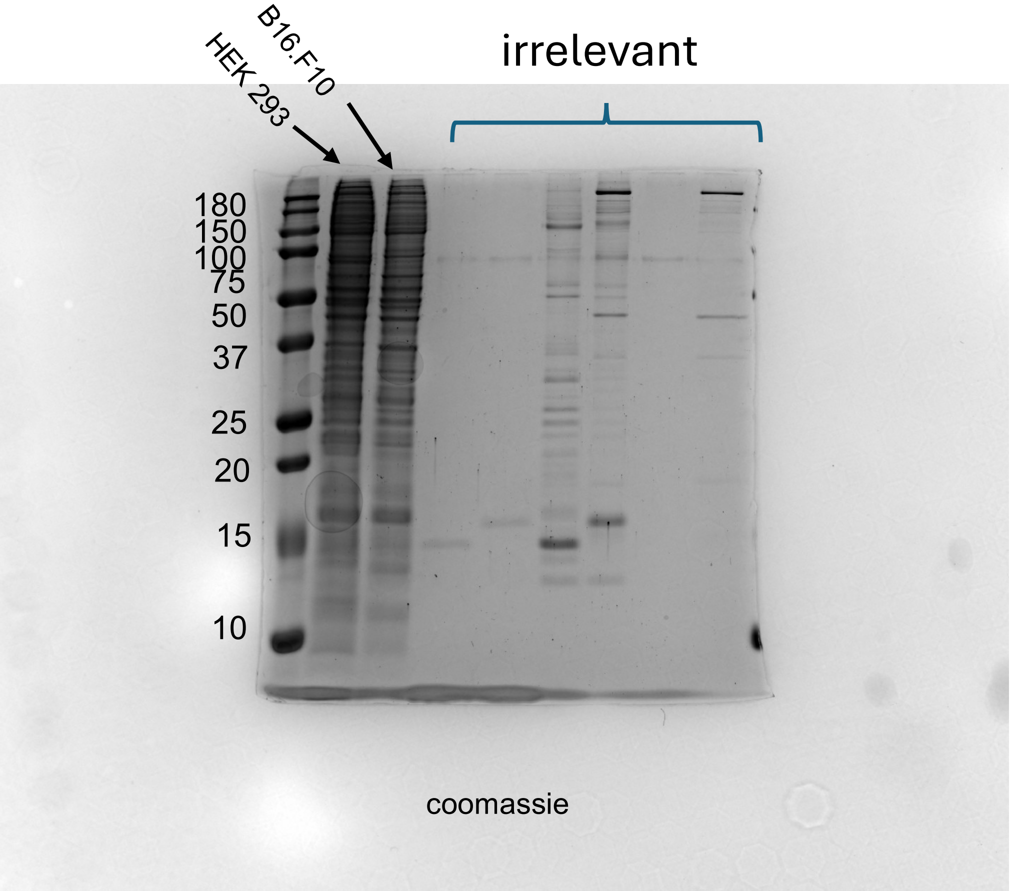

Source Gel, Figure 1A, middle panel

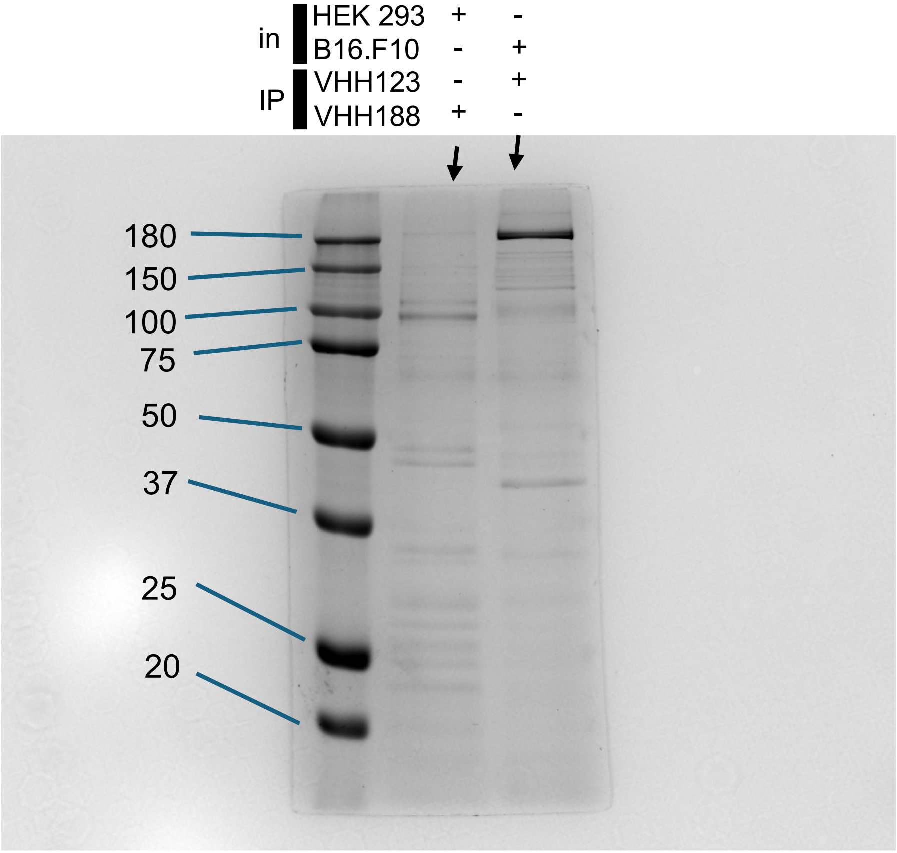

Source Gel, Figure 1A, right panel

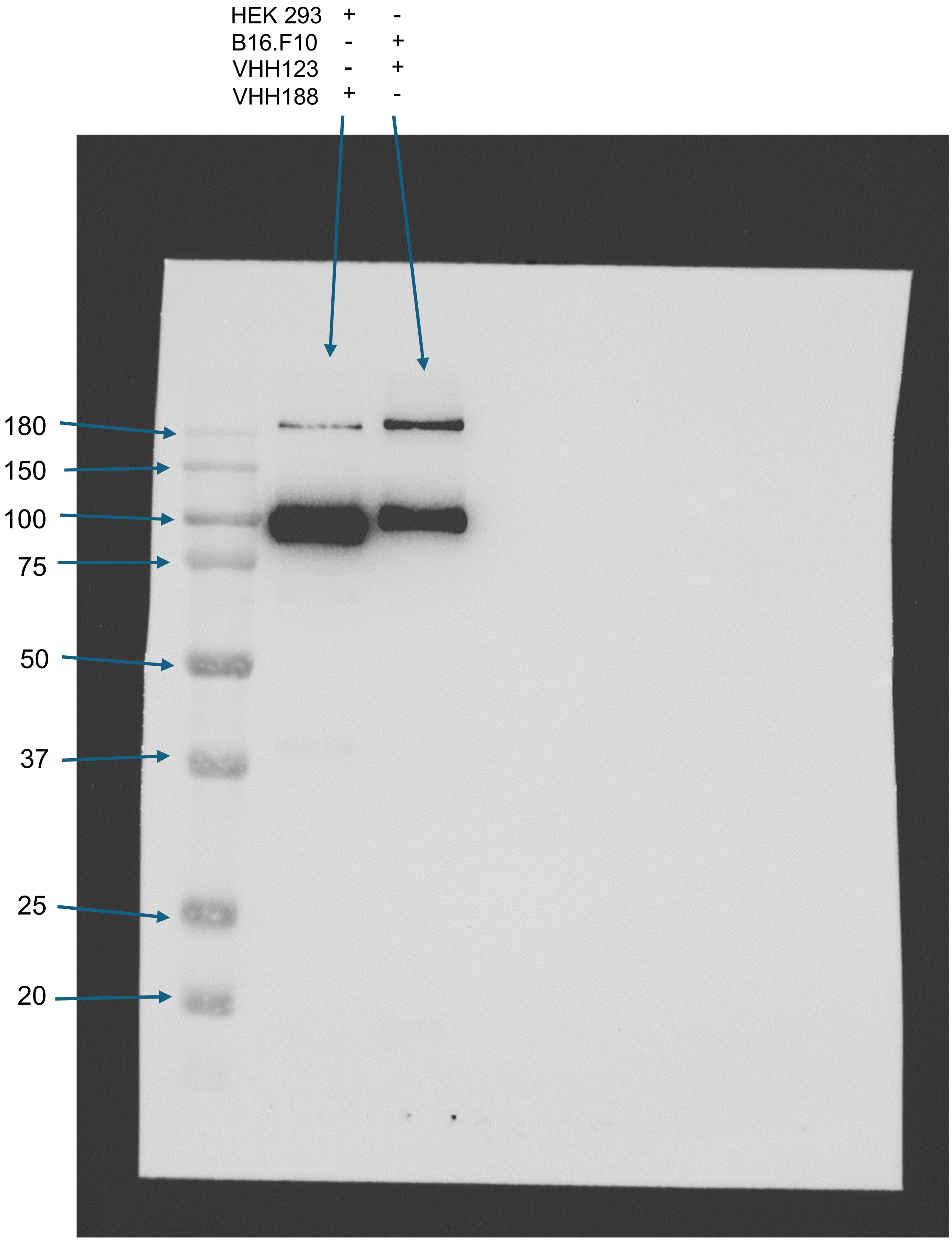

a-Tfr blot

Source Ladder, Figure 1B, HEK293T cells
(mislabelled as '~~29~~B16F10 cells')

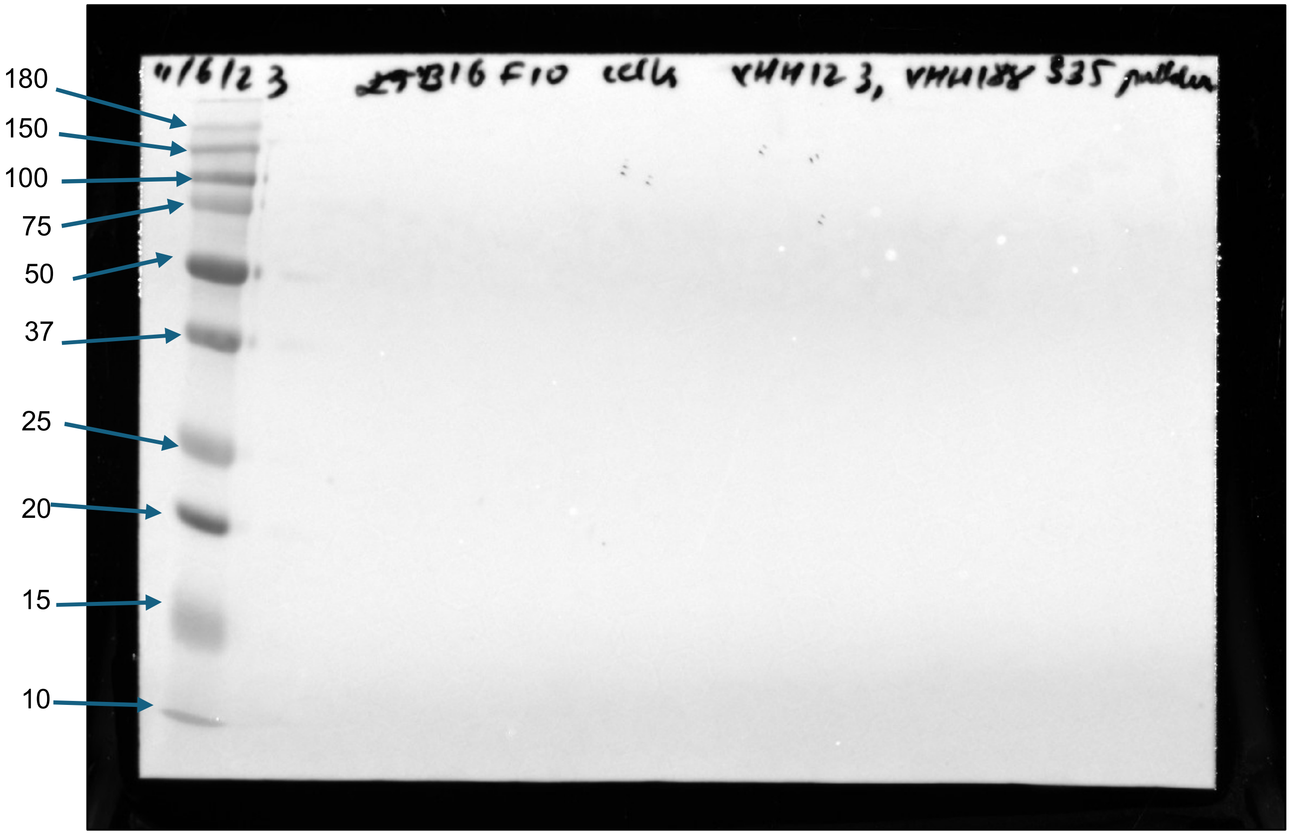

Source Blot, Figure 1B, HEK293T cells

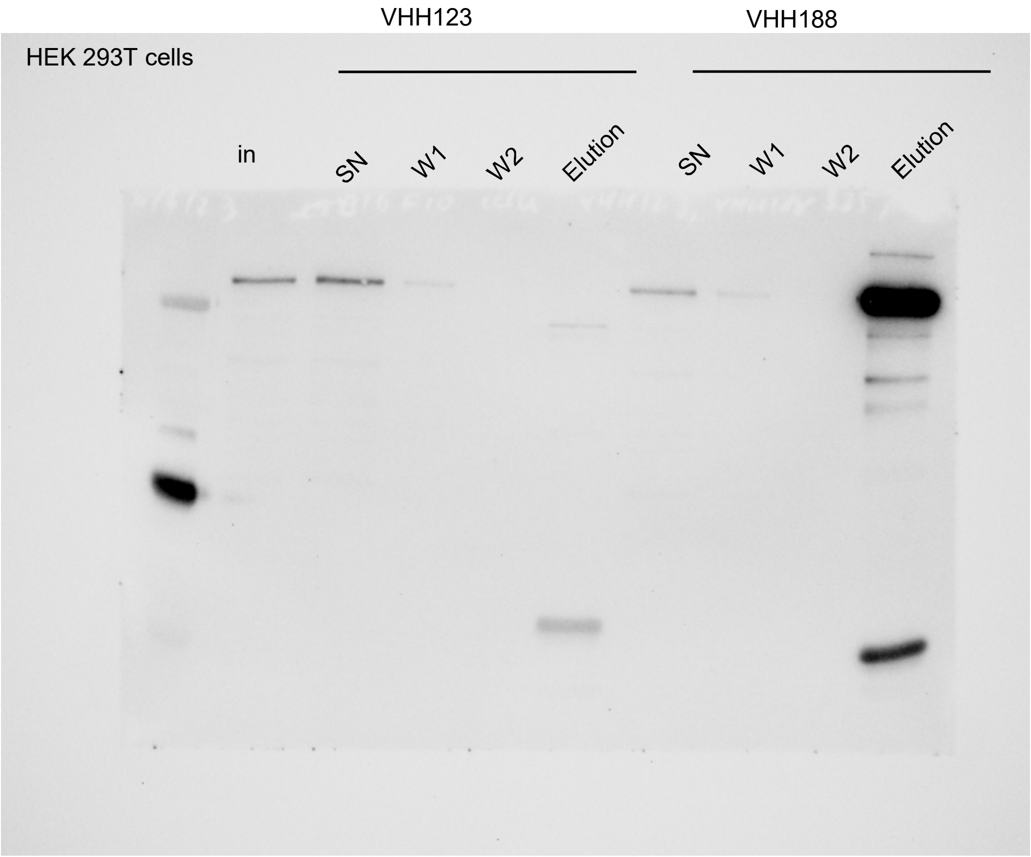

Source Blot, Figure 1B, B16F10 cells
(mislabelled on blot as '293T cells'

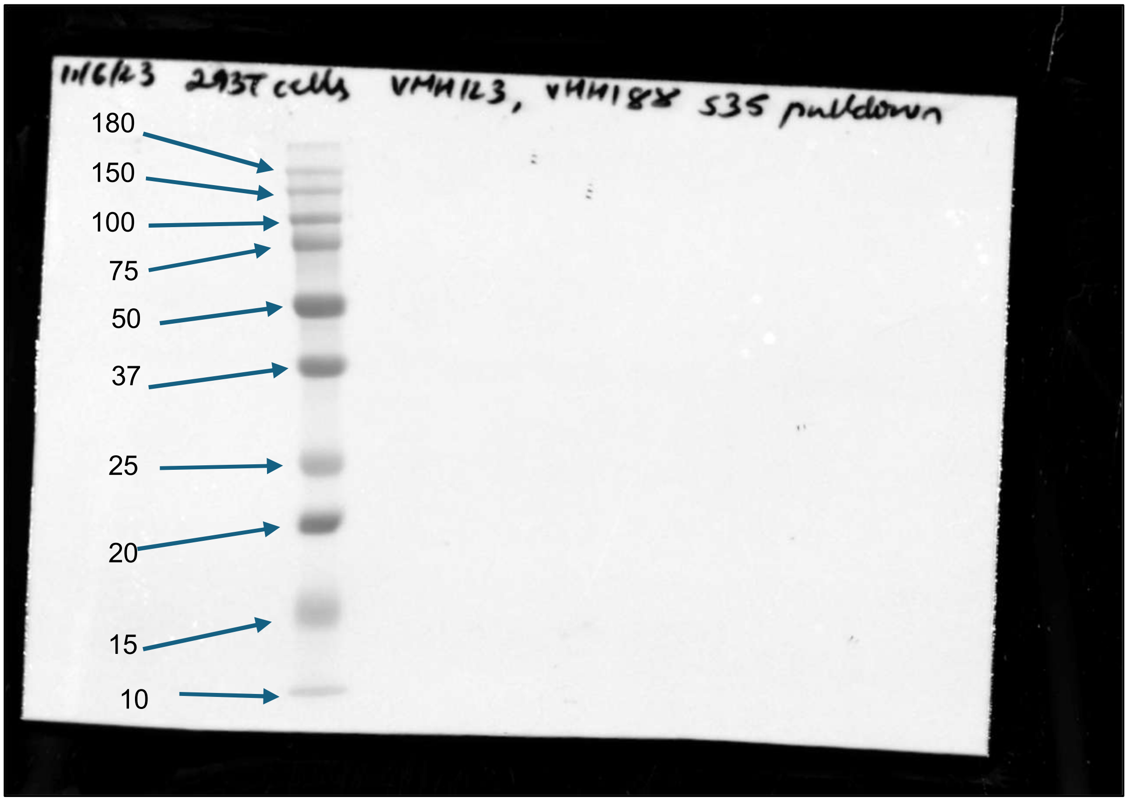

Source Blot, Figure 1B, B16F10 cells

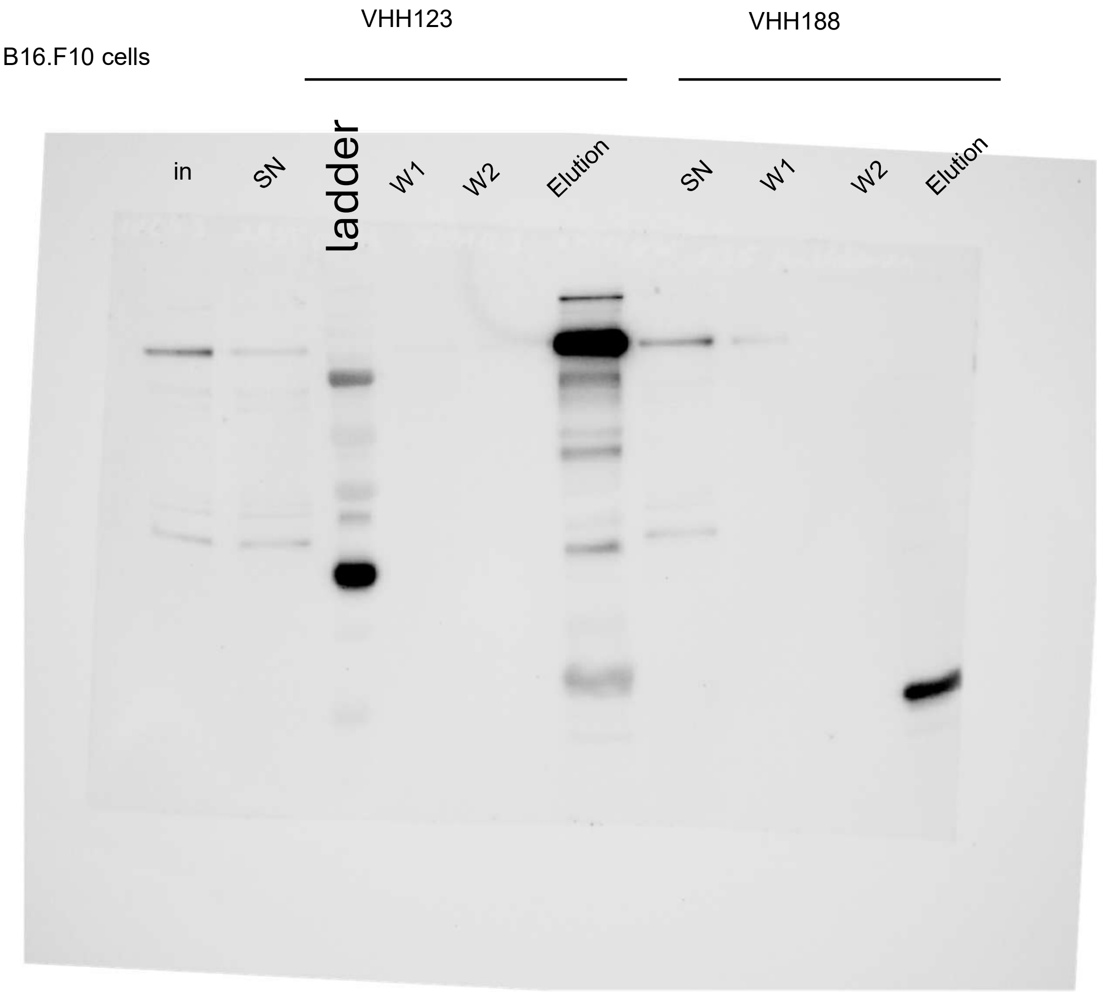

Supplement: Figure 1—source data 1. [file elife-104302-fig1-data1.zip › SOURCE figure 1.pdf]
