# Supplementary figures and images for: A pair of congenic mice for imaging of transplants by positron emission tomography using anti-transferrin receptor nanobodies
Source: eLife. 2025 Aug 18;14:RP104302. doi: 10.7554/eLife.104302 (PMC12360783; doi:10.7554/eLife.104302)

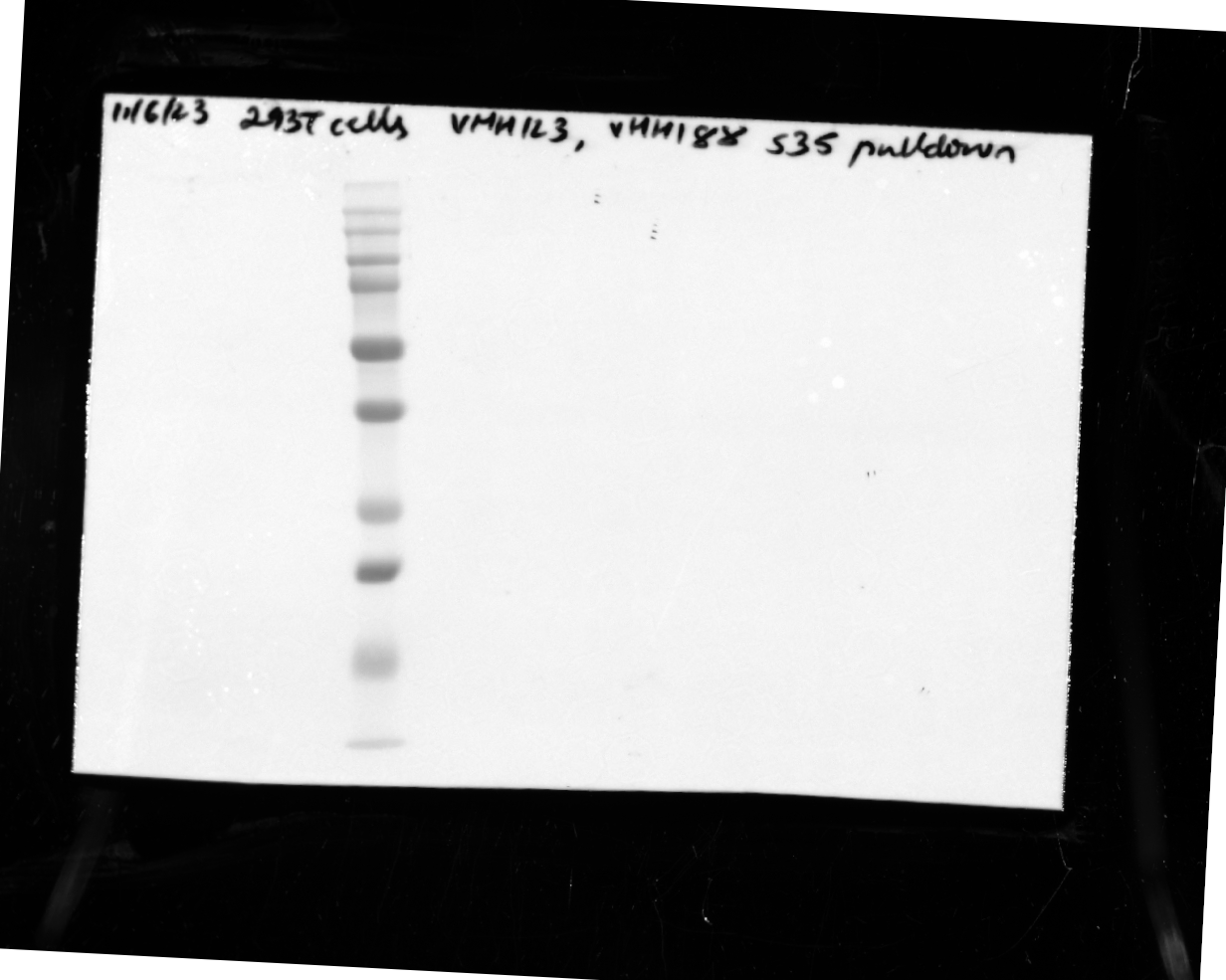

Supplement: Figure 1—source data 2. [file elife-104302-fig1-data2.zip › Figure 1B- HEK293 source ladder.png]

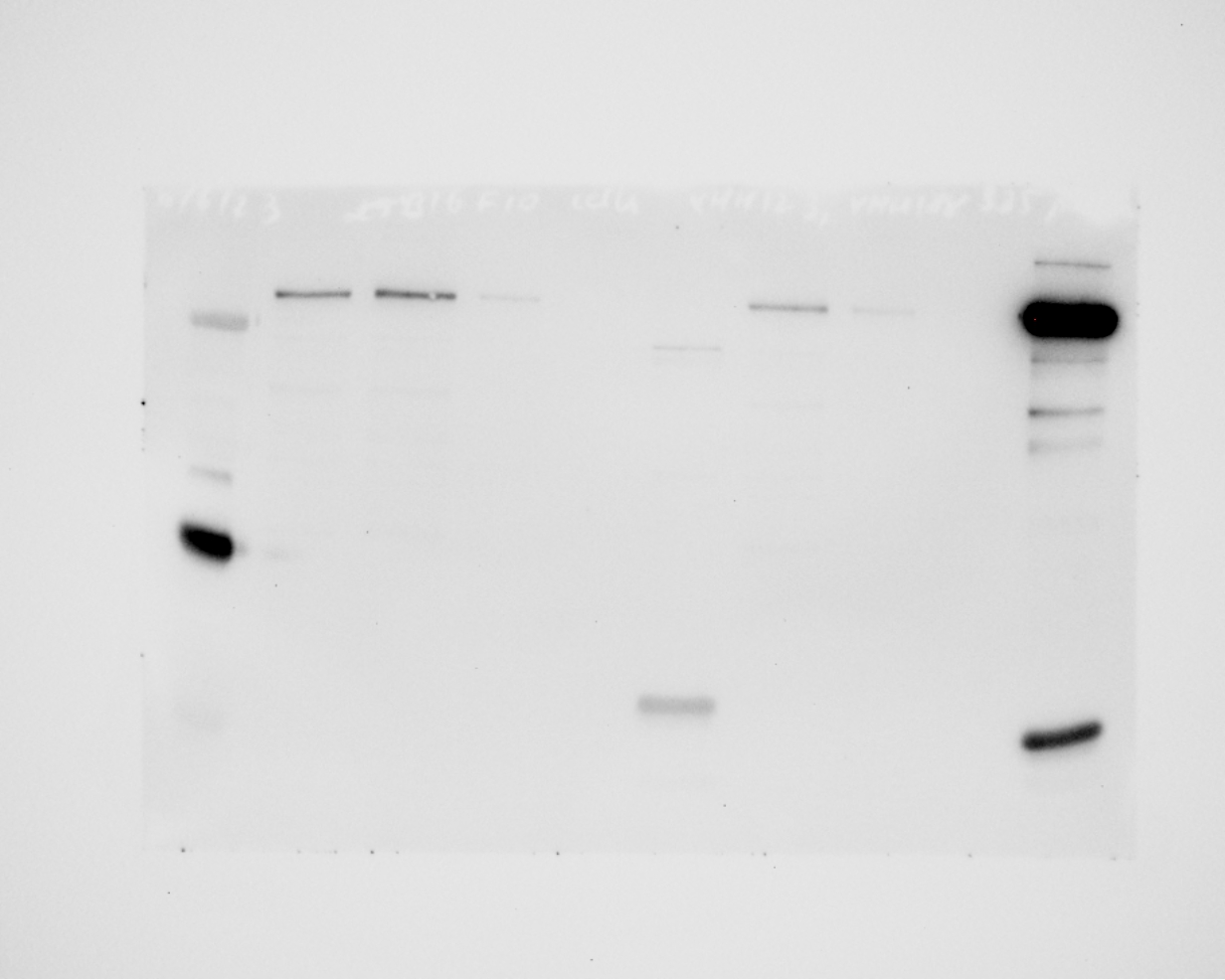

Supplement: Figure 1—source data 2. [file elife-104302-fig1-data2.zip › Figure 1B-B16F10 source blot.jpg]

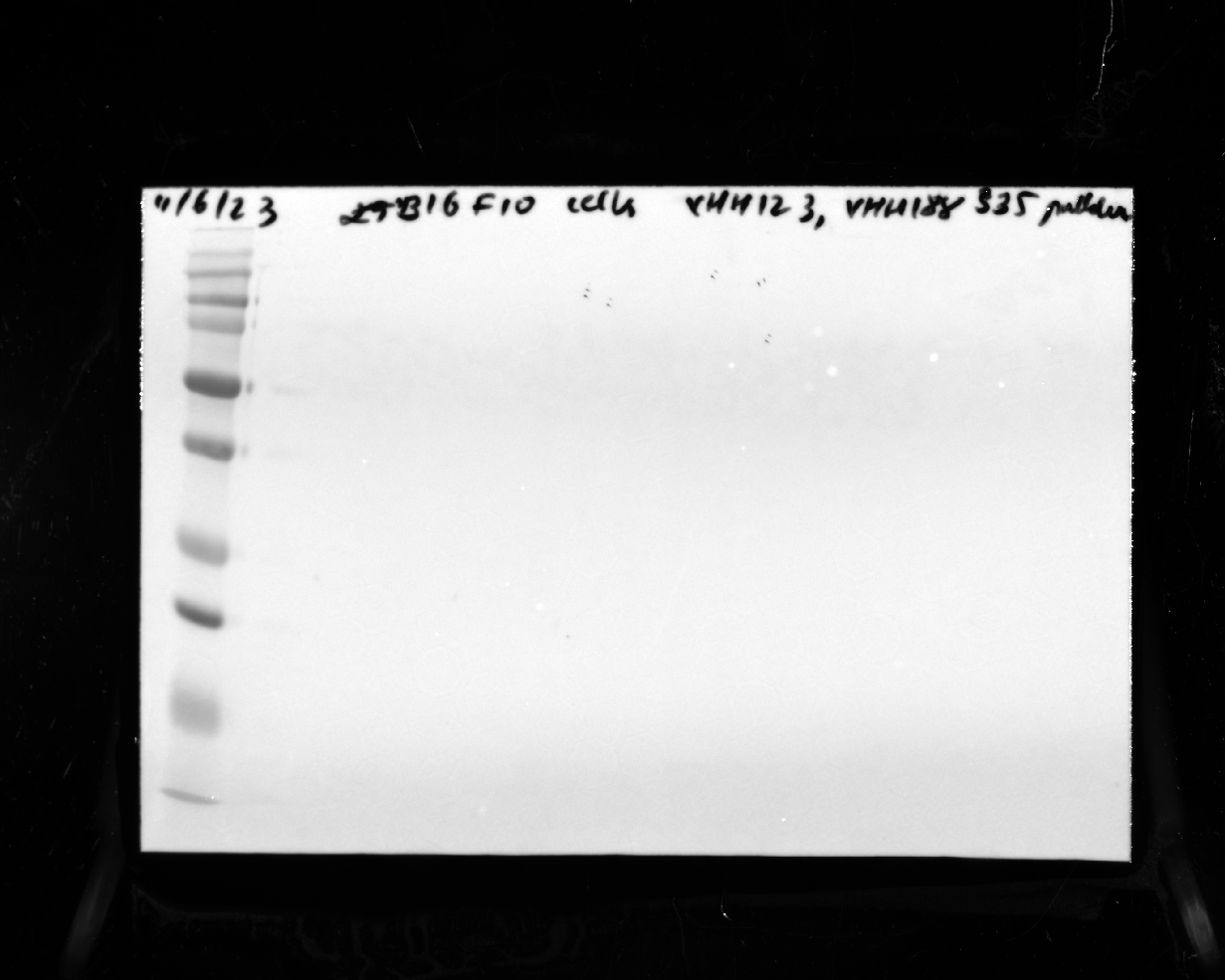

Supplement: Figure 1—source data 2. [file elife-104302-fig1-data2.zip › Figure 1B-B16F10 source ladder.jpg]

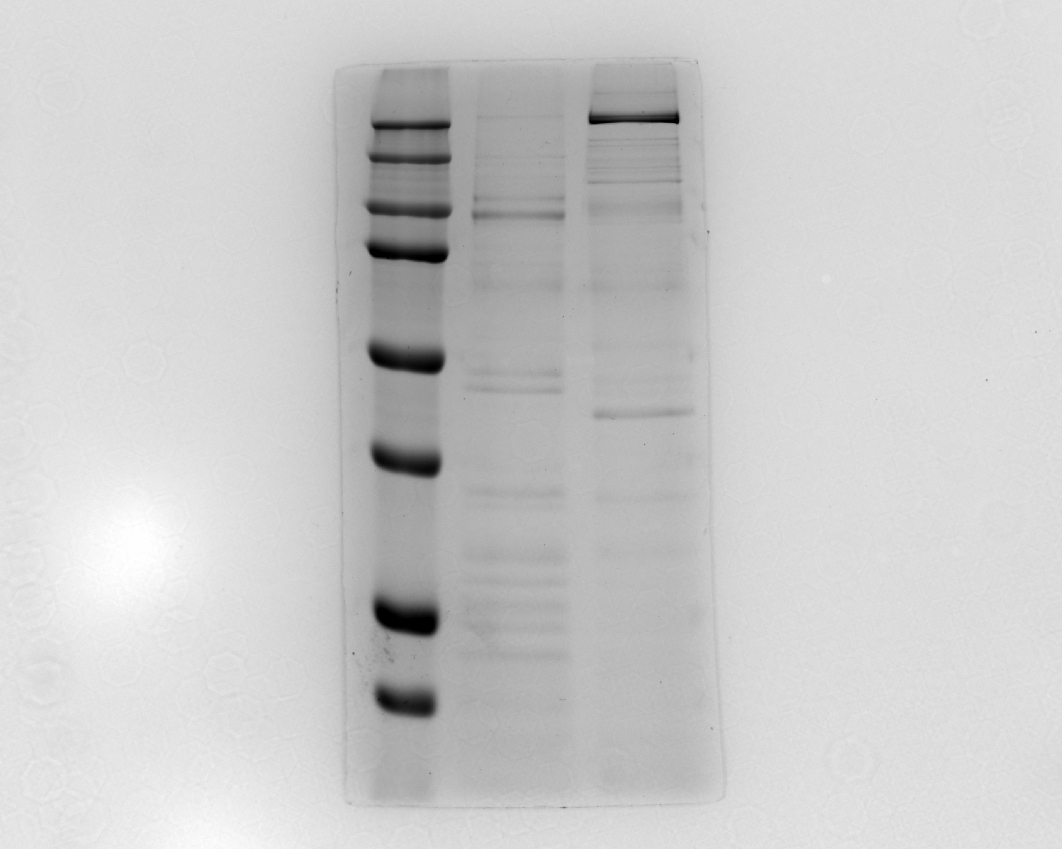

Supplement: Figure 1—source data 2. [file elife-104302-fig1-data2.zip › Figure 1A - middle pannel gel.jpg]

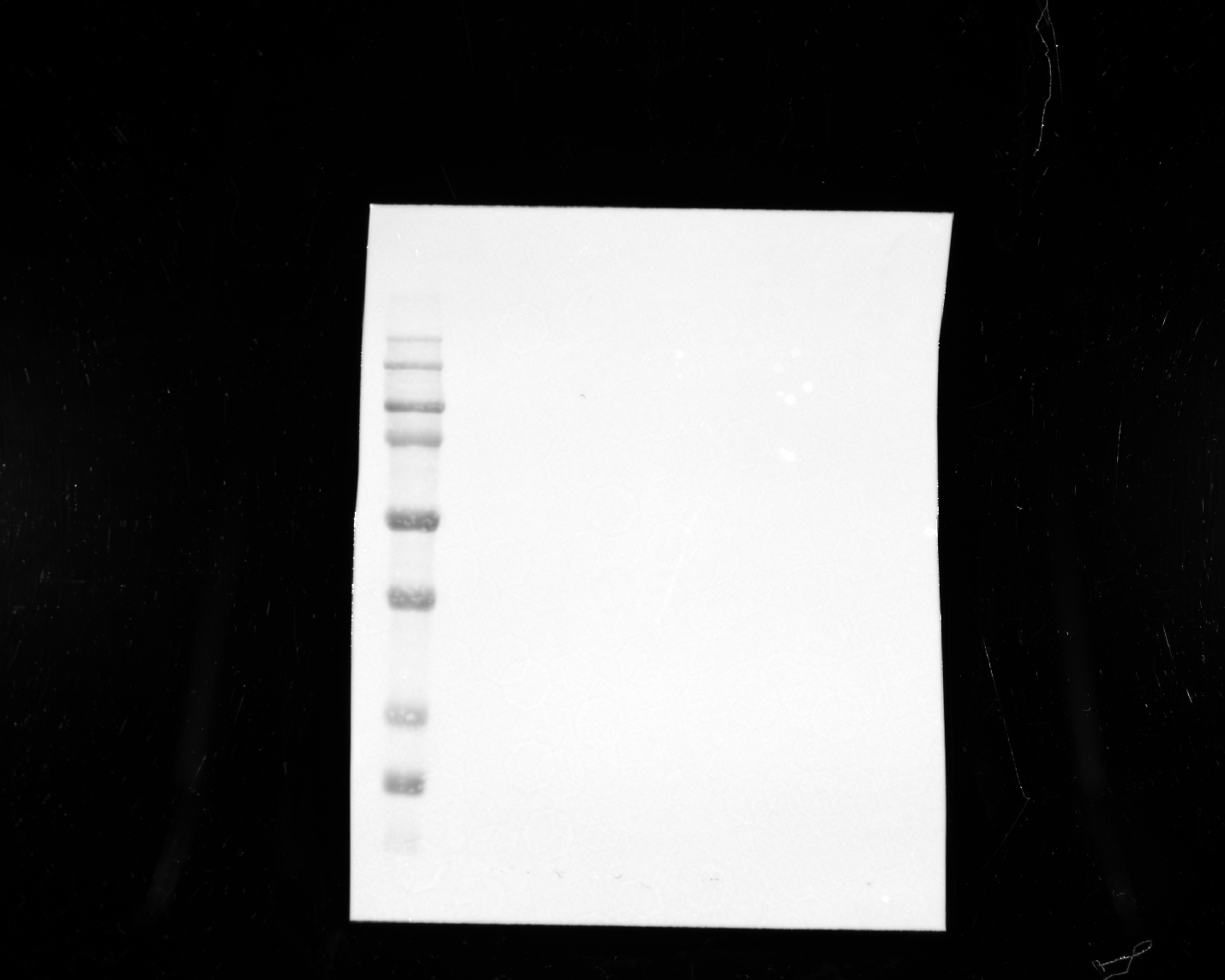

Supplement: Figure 1—source data 2. [file elife-104302-fig1-data2.zip › Figure 1A - right panel - ladder.jpg]

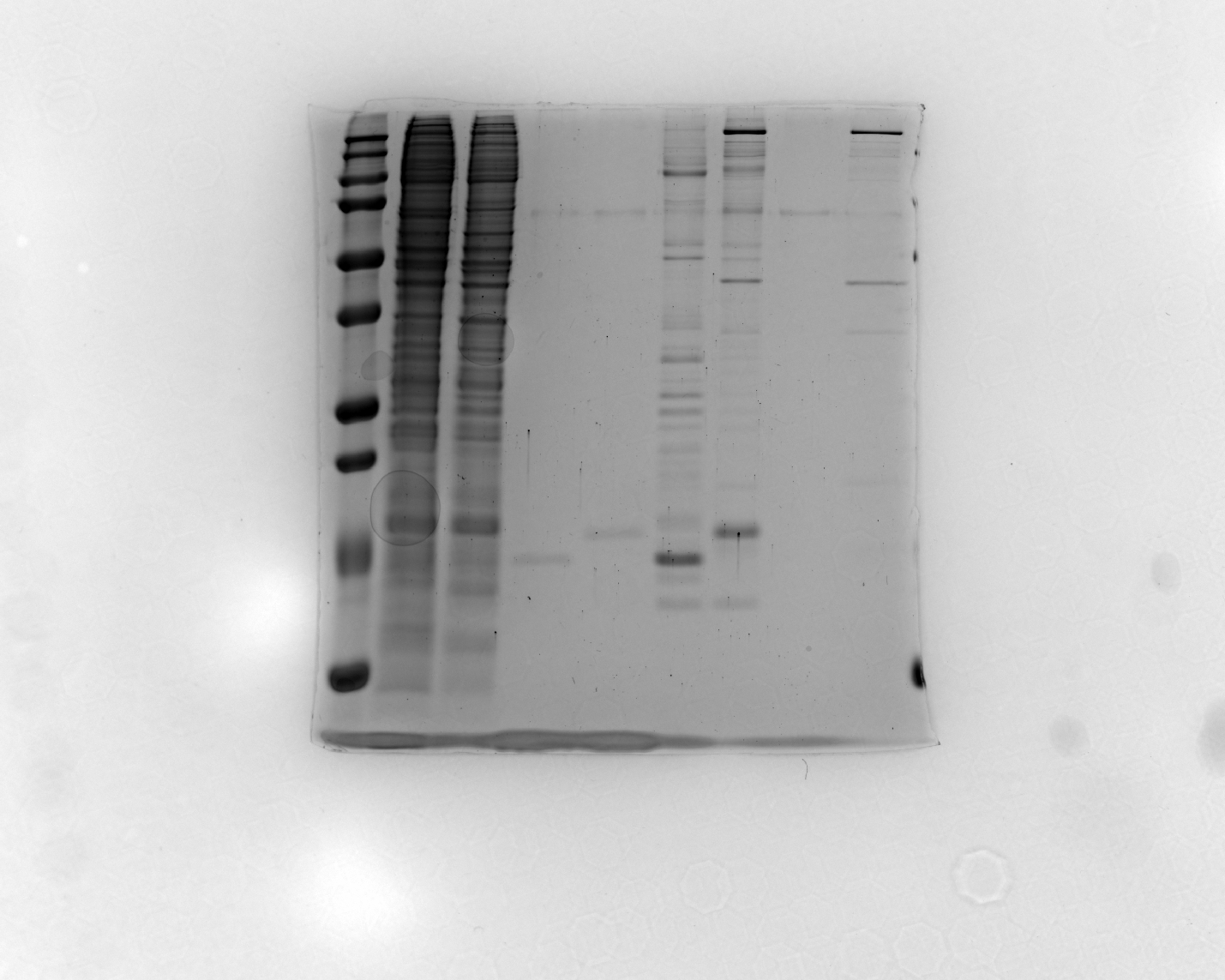

Supplement: Figure 1—source data 2. [file elife-104302-fig1-data2.zip › Figure 1A-left panel.jpg]

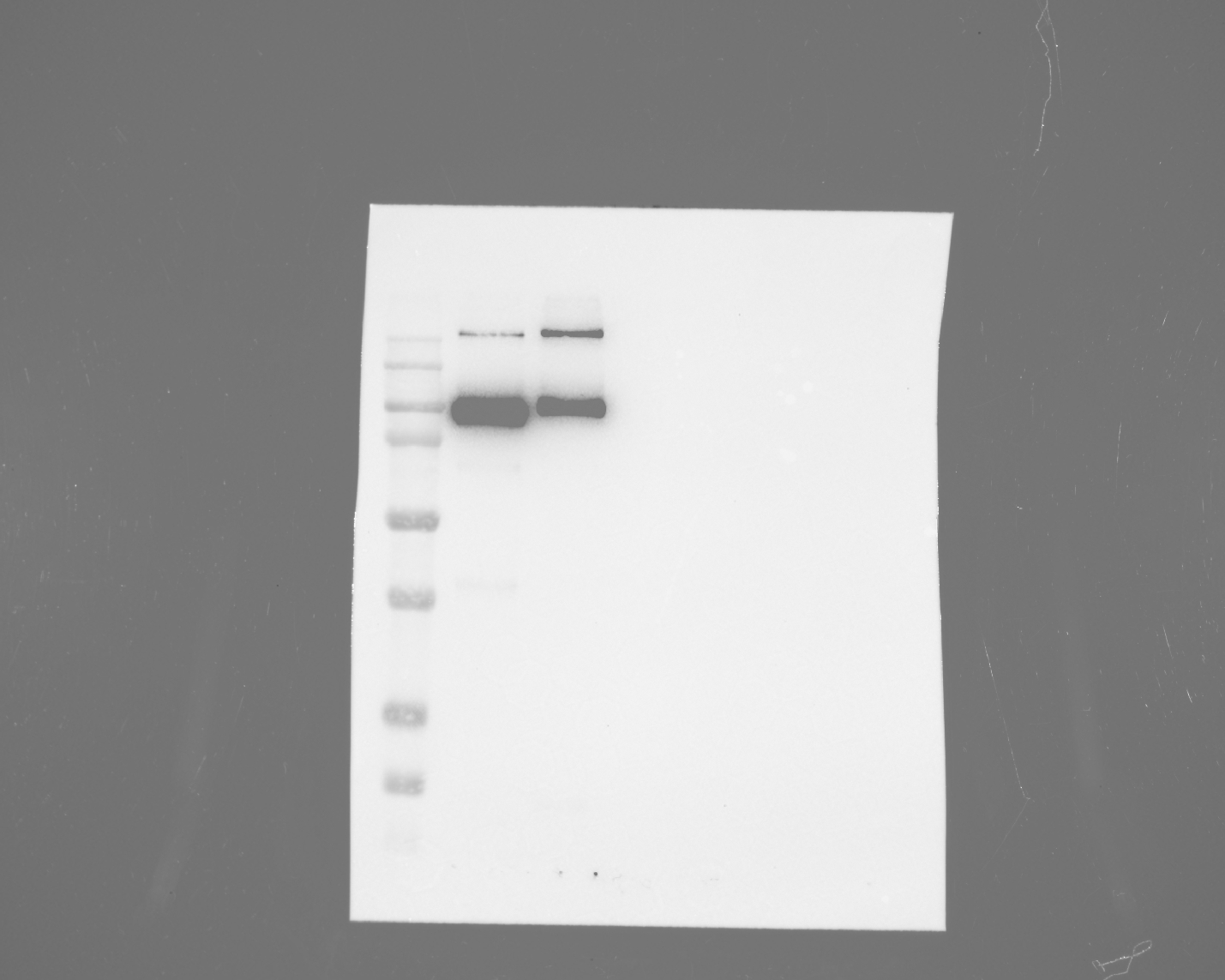

Supplement: Figure 1—source data 2. [file elife-104302-fig1-data2.zip › Figure 1A-right panel - composite.jpg]

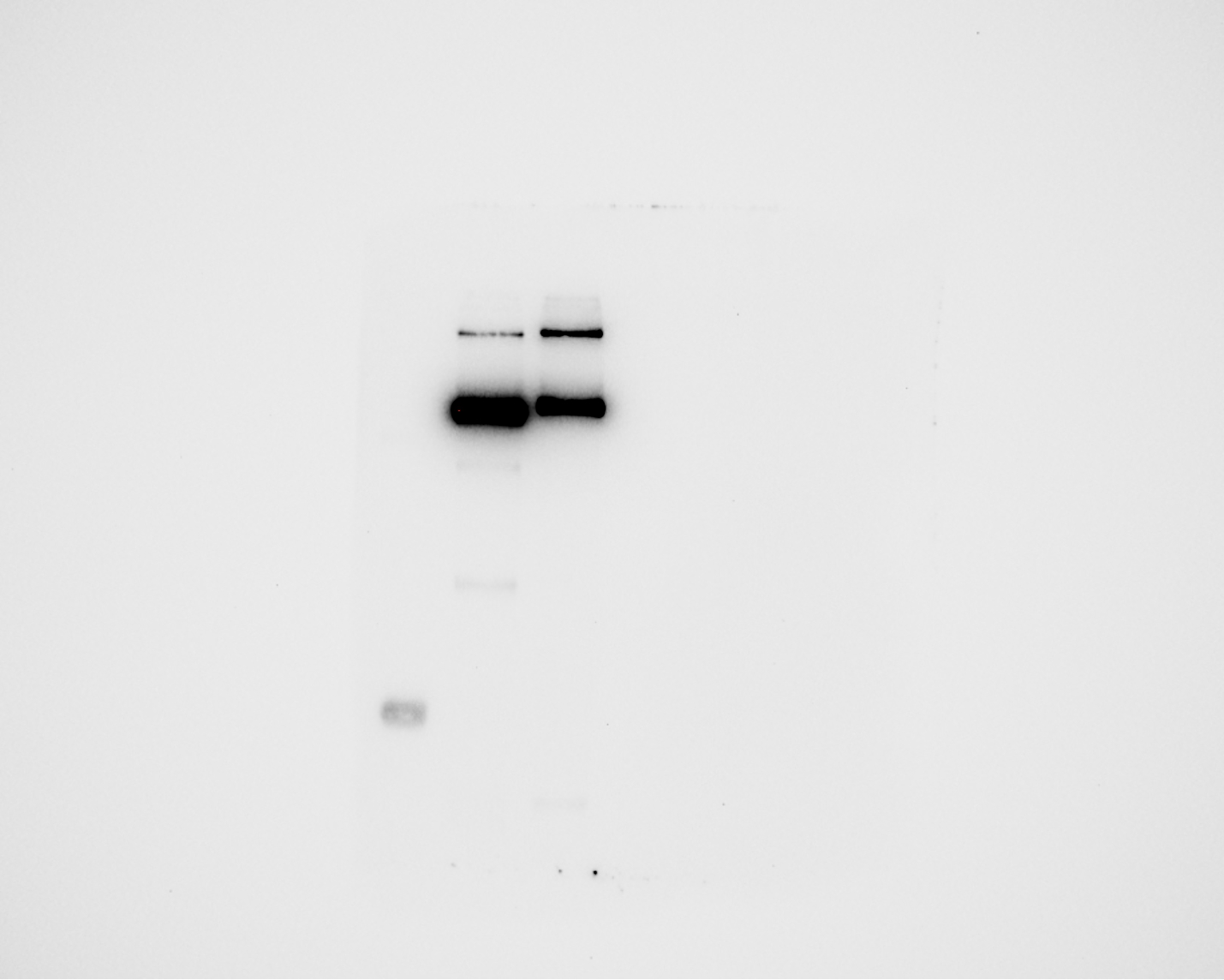

Supplement: Figure 1—source data 2. [file elife-104302-fig1-data2.zip › Figure 1A-rigth panel - blot.jpg]

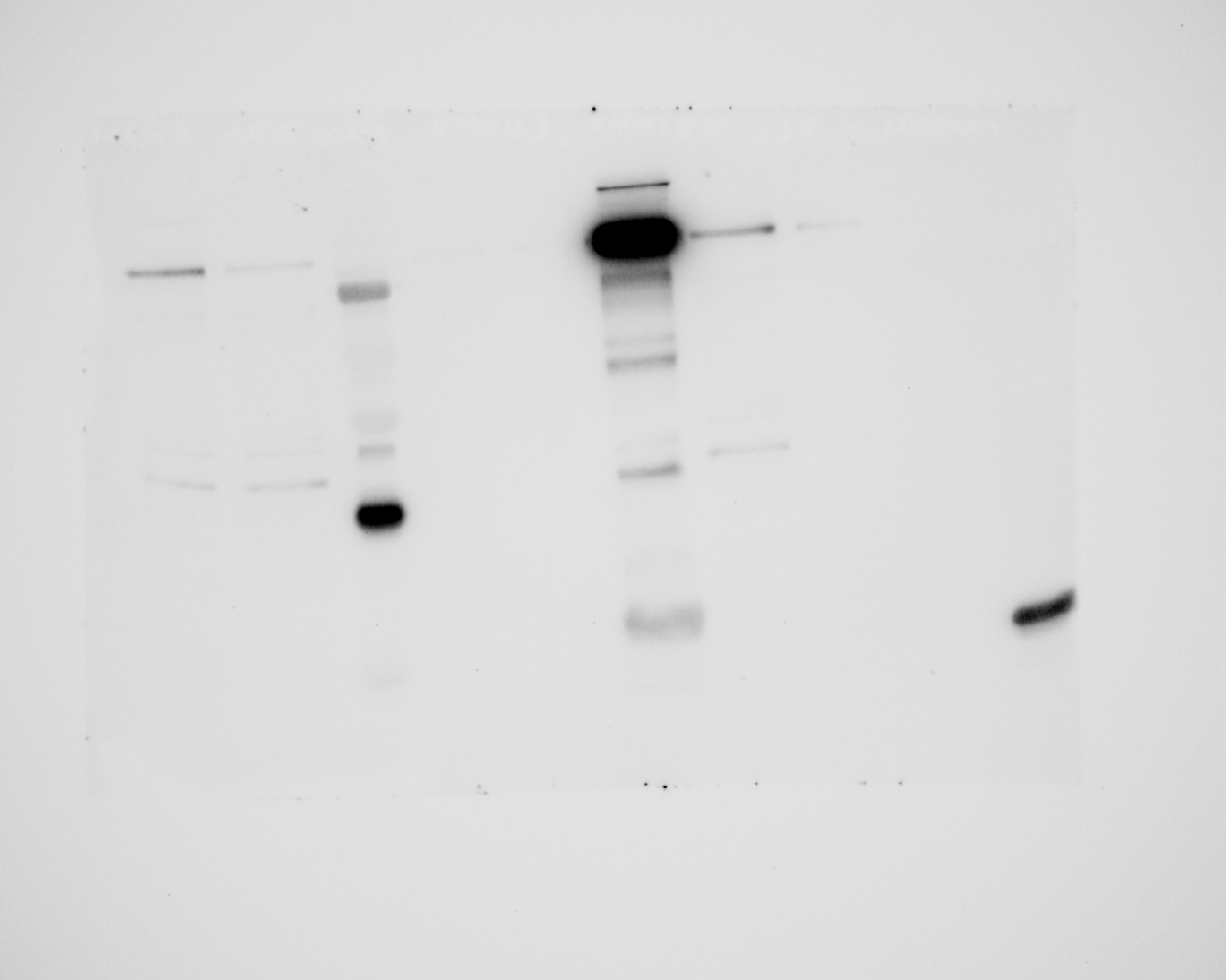

Supplement: Figure 1—source data 2. [file elife-104302-fig1-data2.zip › Figure 1B - HEK293 source blot.jpg]

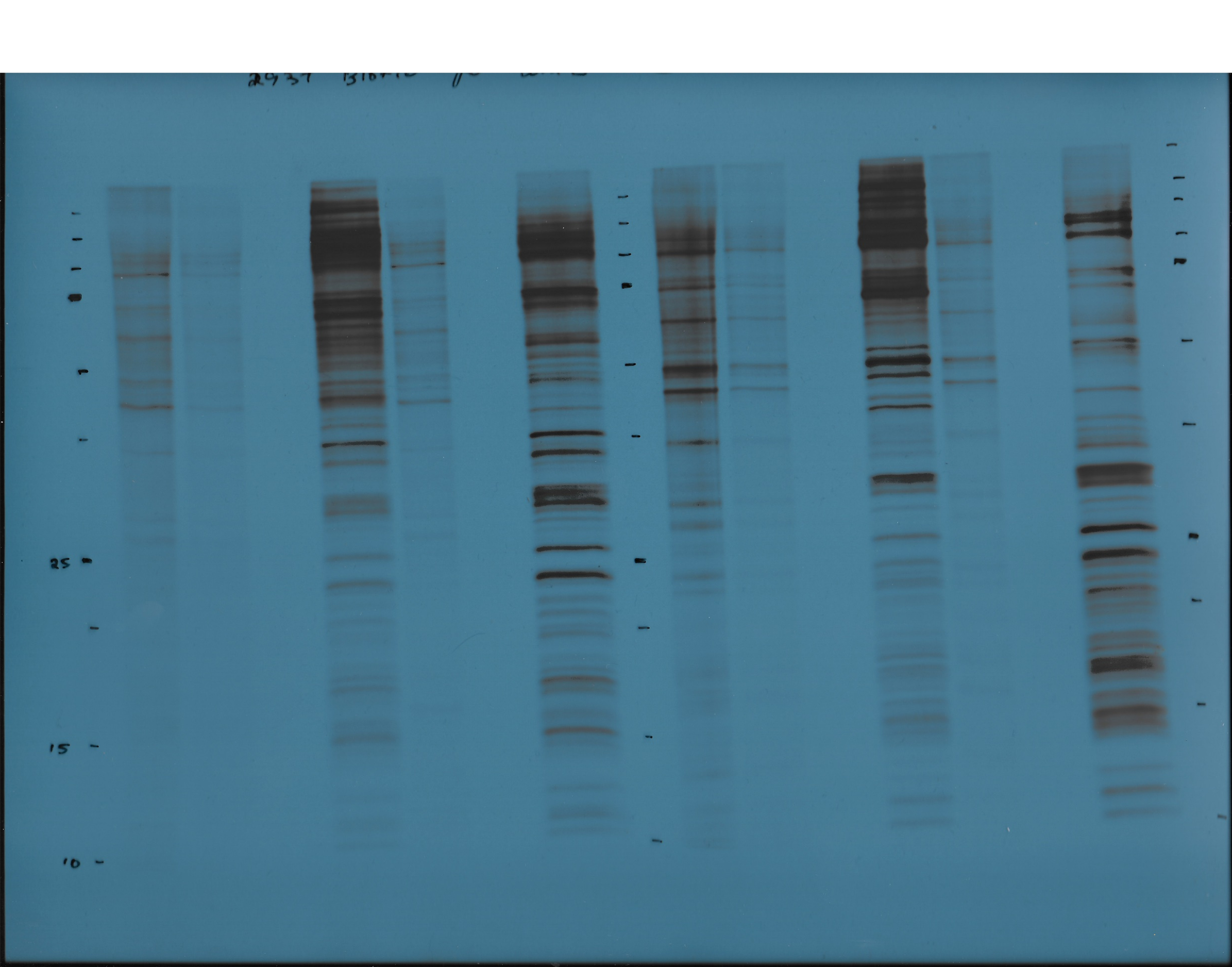

Supplement: Figure 1—figure supplement 1—source data 2. [file elife-104302-fig1-figsupp1-data2.zip › Figure 1-figure supplemental 1 - raw scan of photofilm.png]

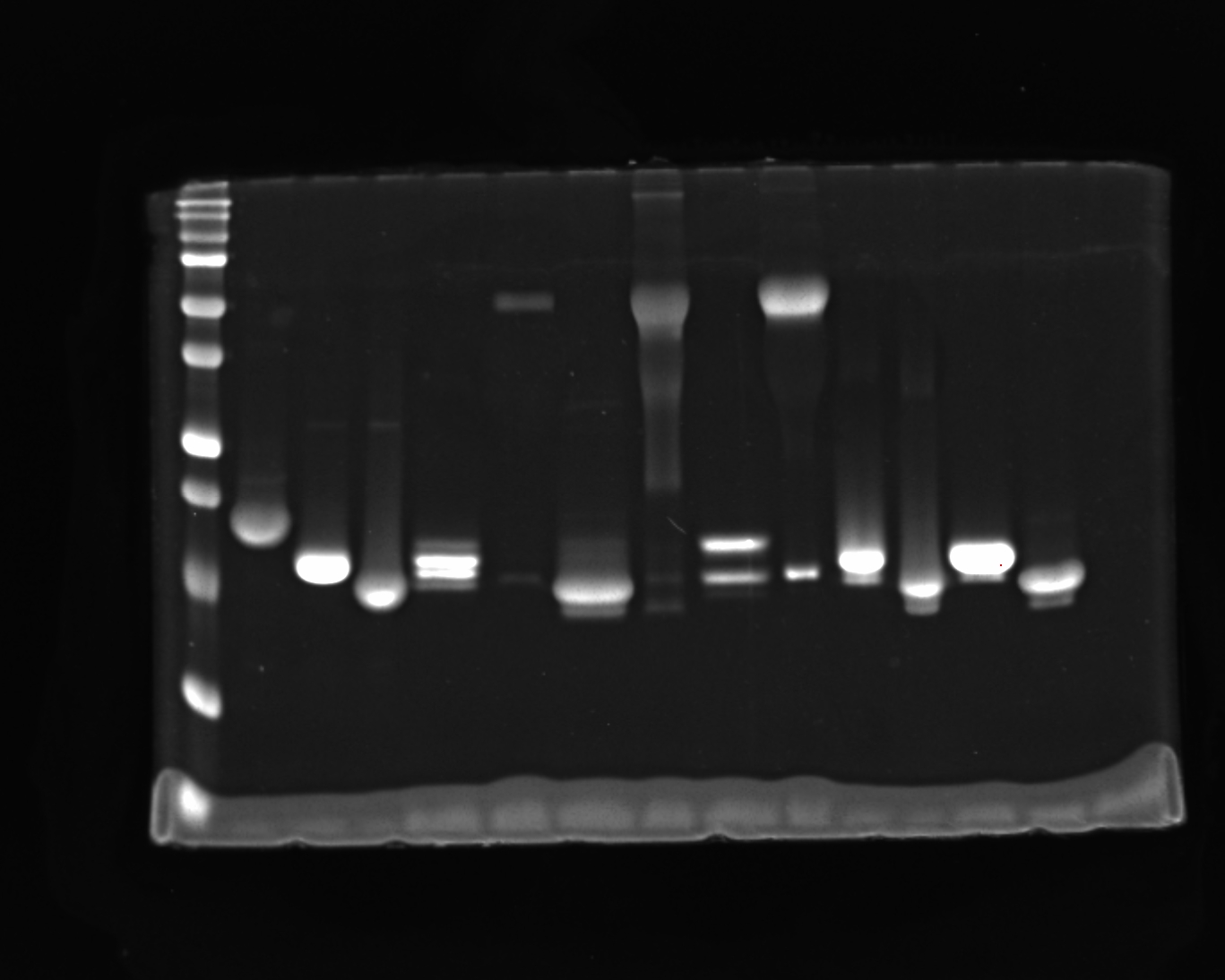

Supplement: Figure 2—figure supplement 2—source data 2. [file elife-104302-fig2-figsupp2-data2.zip › Figure 2-figure supplement 2 - raw gel image.jpg]
