# Supplementary material for: A pair of congenic mice for imaging of transplants by positron emission tomography using anti-transferrin receptor nanobodies
Source: eLife. 2025 Aug 18;14:RP104302. doi: 10.7554/eLife.104302 (PMC12360783; doi:10.7554/eLife.104302)

# Source autoradiograph, figure 1-figure supplement 1

S35 labeled cell lysate pulldown using biotinylated VHH123 and VHH188

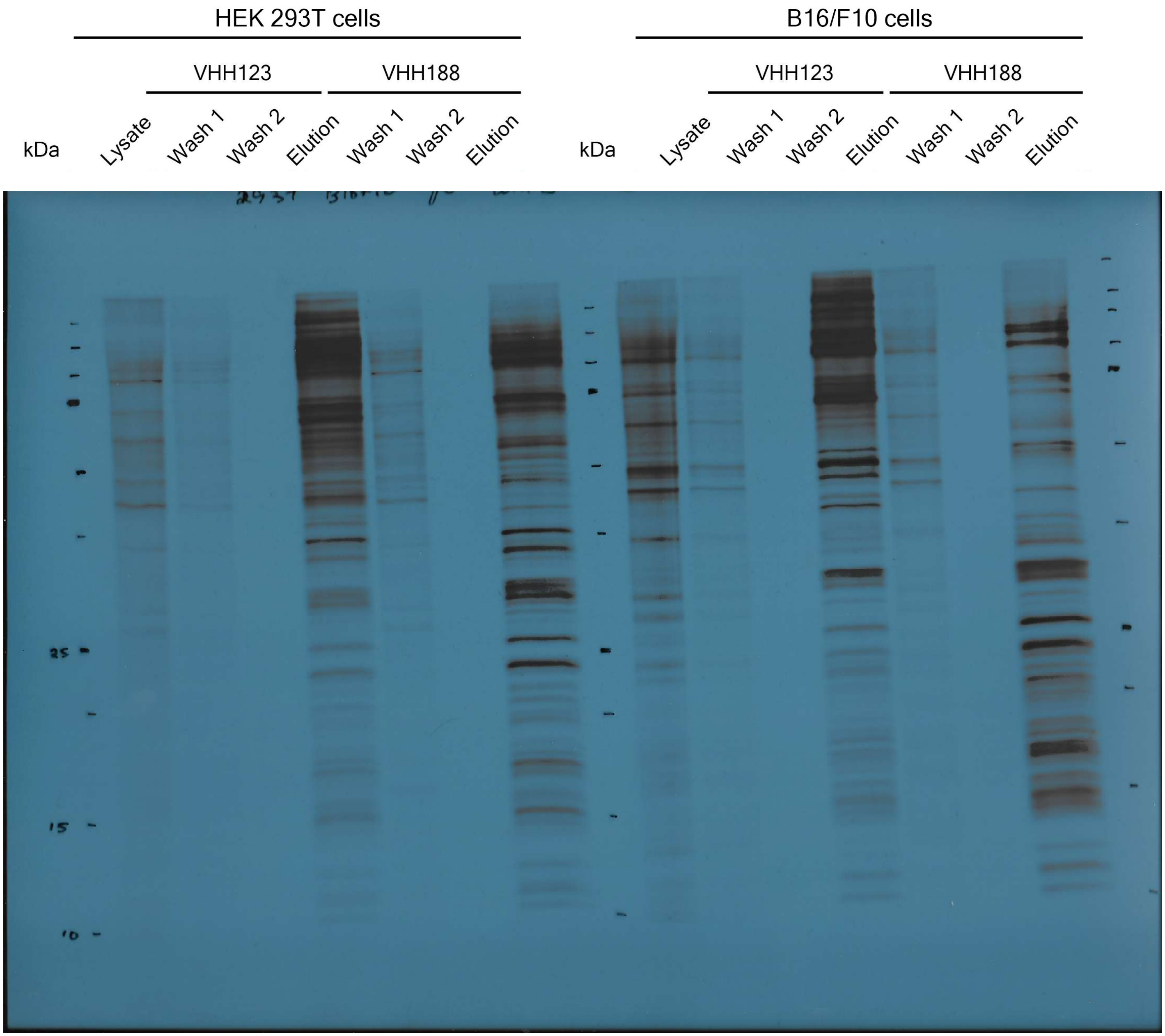

Supplement: Figure 1—figure supplement 1—source data 1. [file elife-104302-fig1-figsupp1-data1.zip › SOURCE figure 1-figure supplement 1.pdf]
