# Supplementary material for: A pair of congenic mice for imaging of transplants by positron emission tomography using anti-transferrin receptor nanobodies
Source: eLife. 2025 Aug 18;14:RP104302. doi: 10.7554/eLife.104302 (PMC12360783; doi:10.7554/eLife.104302)
Supplement: Figure 2—figure supplement 2—source data 1. [file elife-104302-fig2-figsupp2-data1.zip › SOURCE figure 2-figure supplement2.pdf]

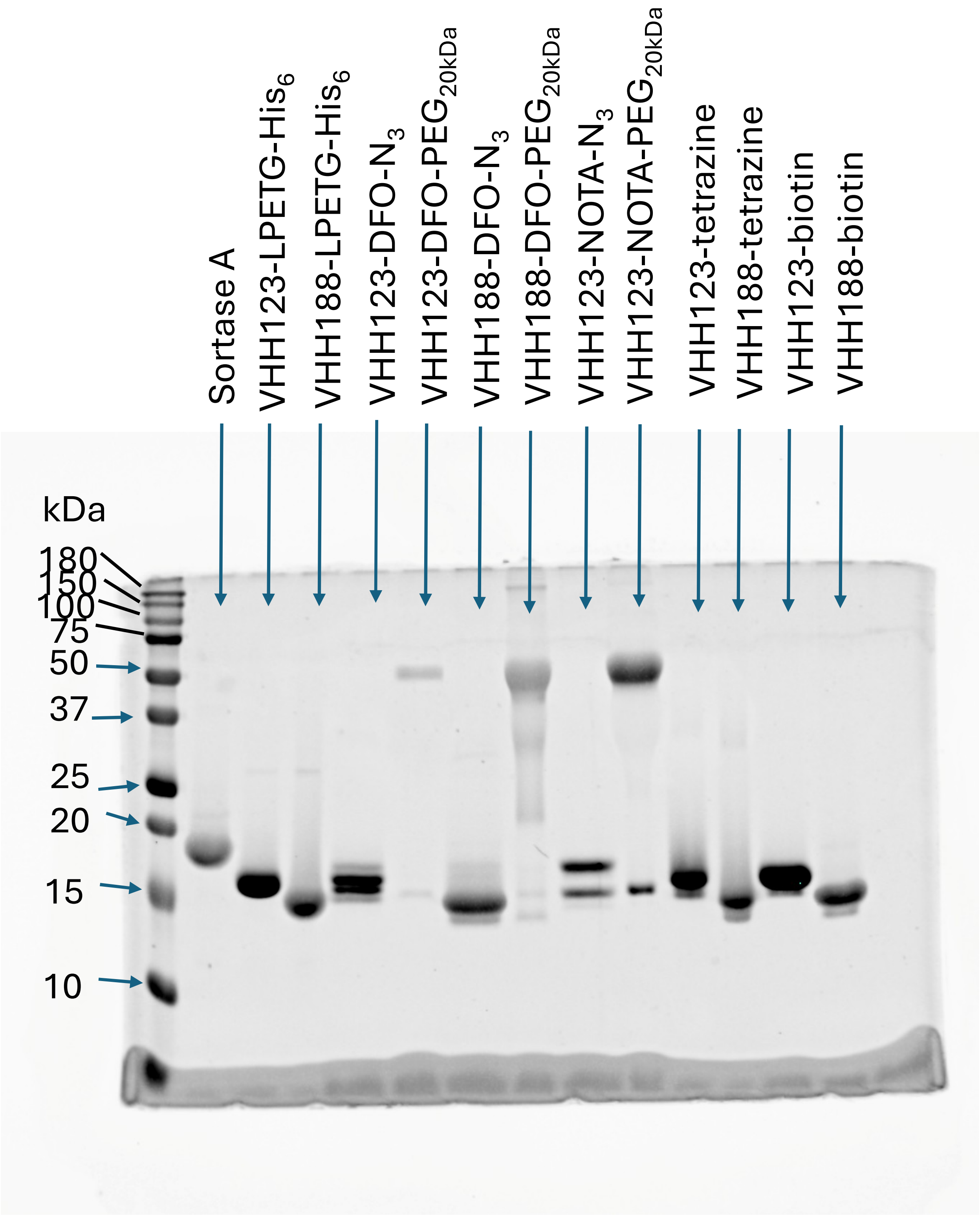

Coomassie stained gel picture

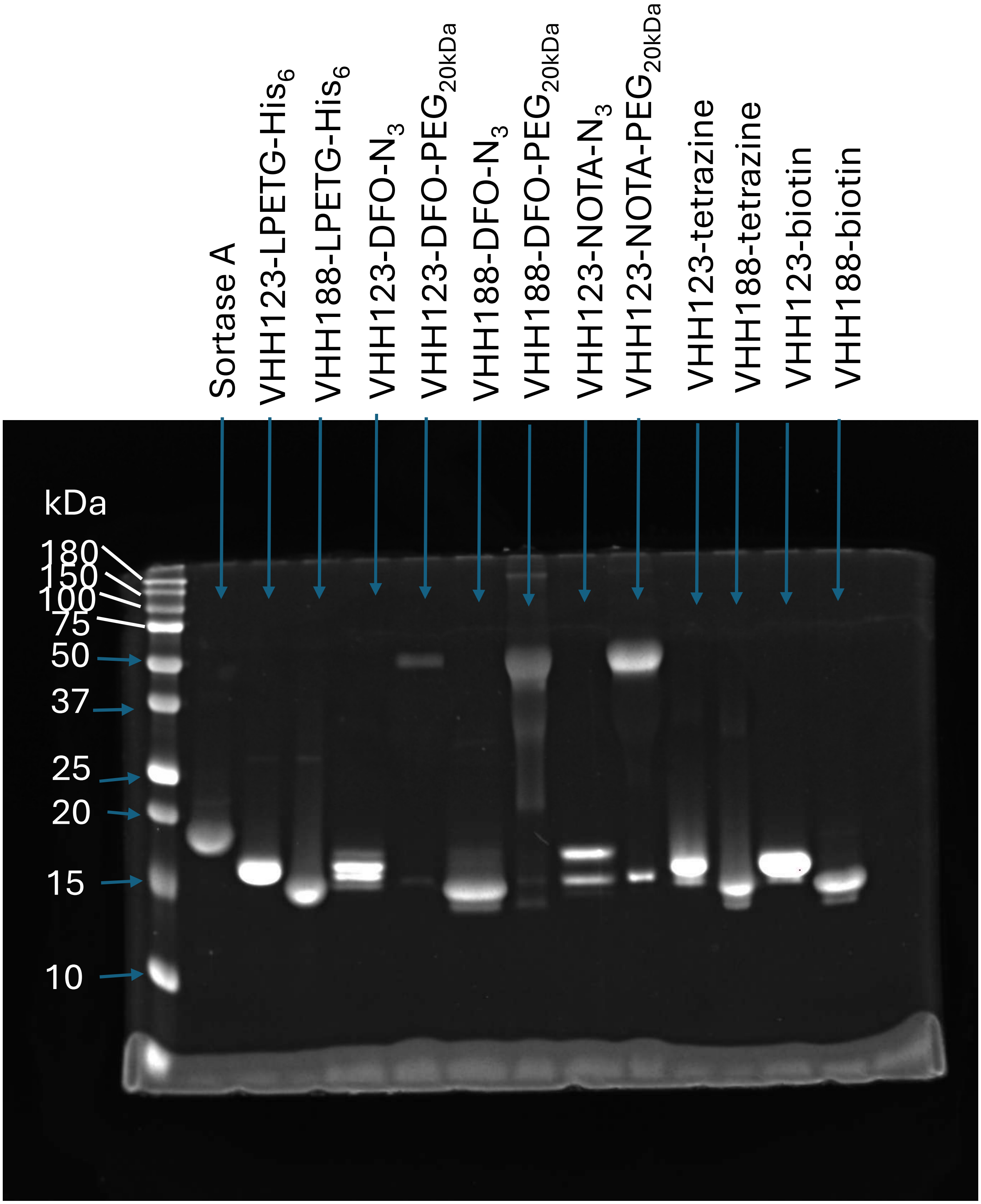

Original file was acquired using a Black Tray and Far Red Epi-light with a BioRad Chemidoc imager.
The image was then inverted (black <-> white) to produce the picture of Figure 2-figure supplement 2
