# Supplementary material for: A pair of congenic mice for imaging of transplants by positron emission tomography using anti-transferrin receptor nanobodies
Source: eLife. 2025 Aug 18;14:RP104302. doi: 10.7554/eLife.104302 (PMC12360783; doi:10.7554/eLife.104302)
Supplement: Figure 2—figure supplement 2—source data 3. [file elife-104302-fig2-figsupp2-data3.zip › LC-MS masses.pptx]

## Slide 1
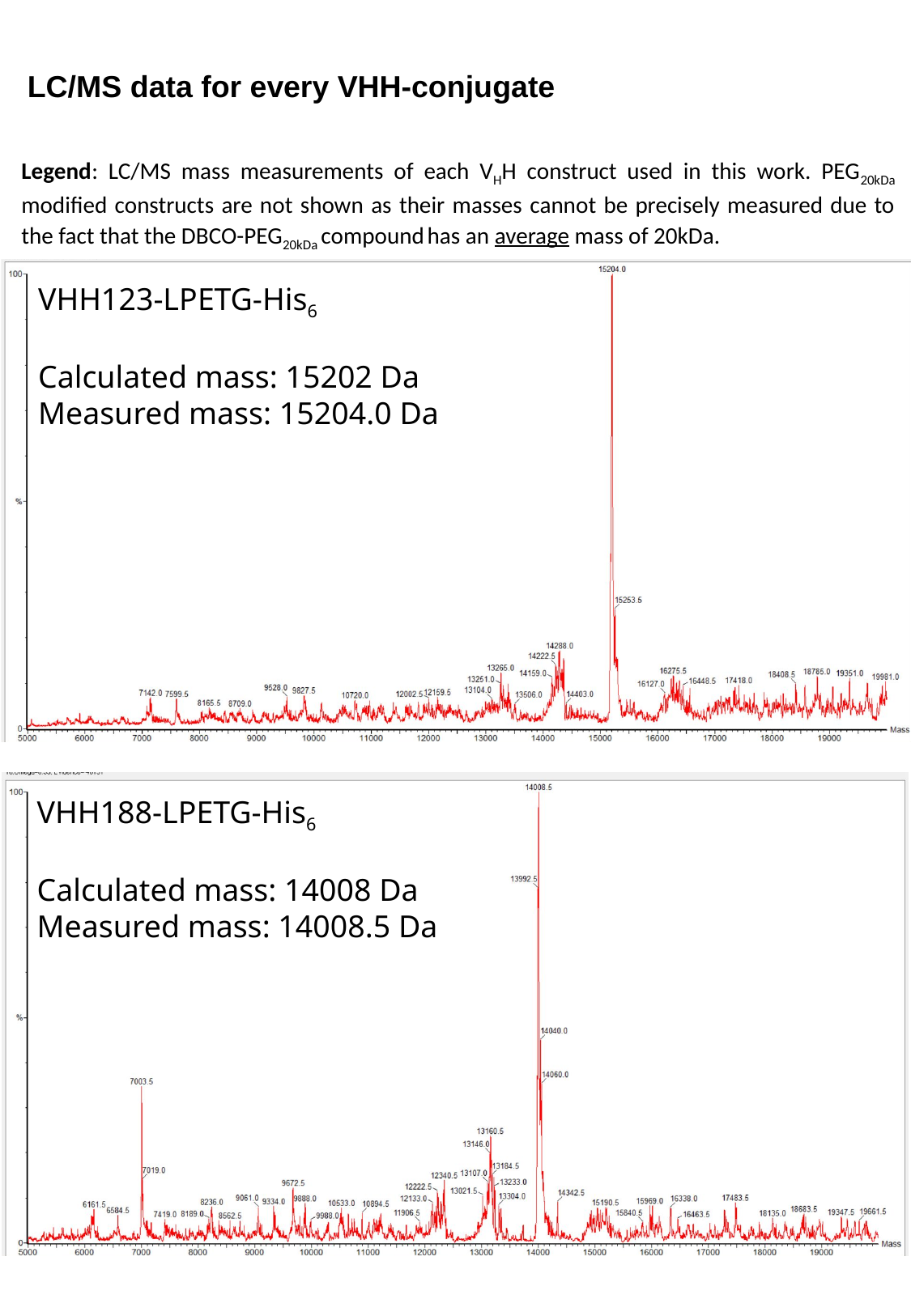

LC/MS data for every VHH-conjugate
Legend: LC/MS mass measurements of each VHH construct used in this work. PEG20kDa modified constructs are not shown as their masses cannot be precisely measured due to the fact that the DBCO-PEG20kDa compound has an average mass of 20kDa.
VHH123-LPETG-His6
Calculated mass: 15202 Da
Measured mass: 15204.0 Da
VHH188-LPETG-His6
Calculated mass: 14008 Da
Measured mass: 14008.5 Da

## Slide 2
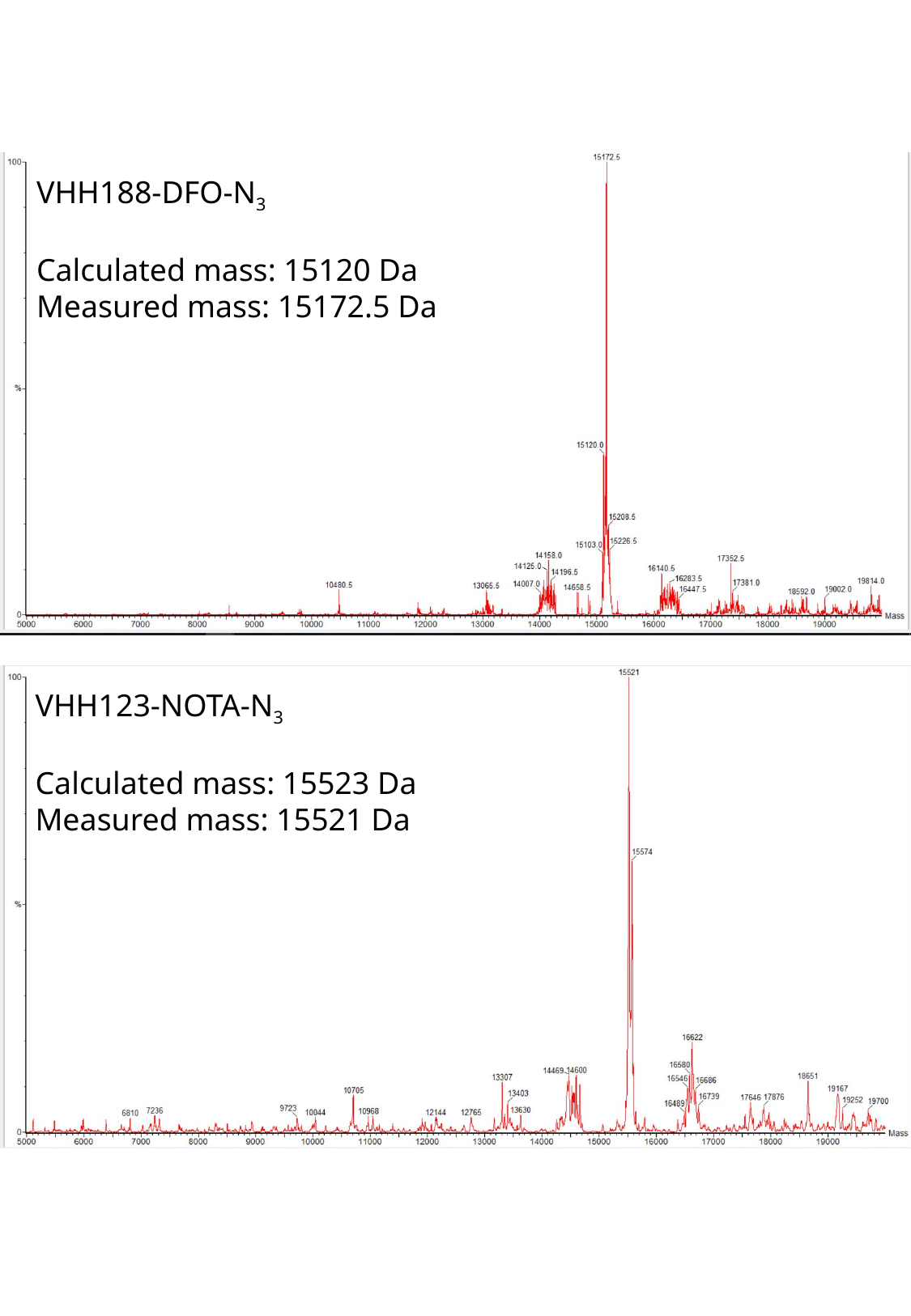

VHH188-DFO-N3
Calculated mass: 15120 Da
Measured mass: 15172.5 Da
VHH123-NOTA-N3
Calculated mass: 15523 Da
Measured mass: 15521 Da

## Slide 3
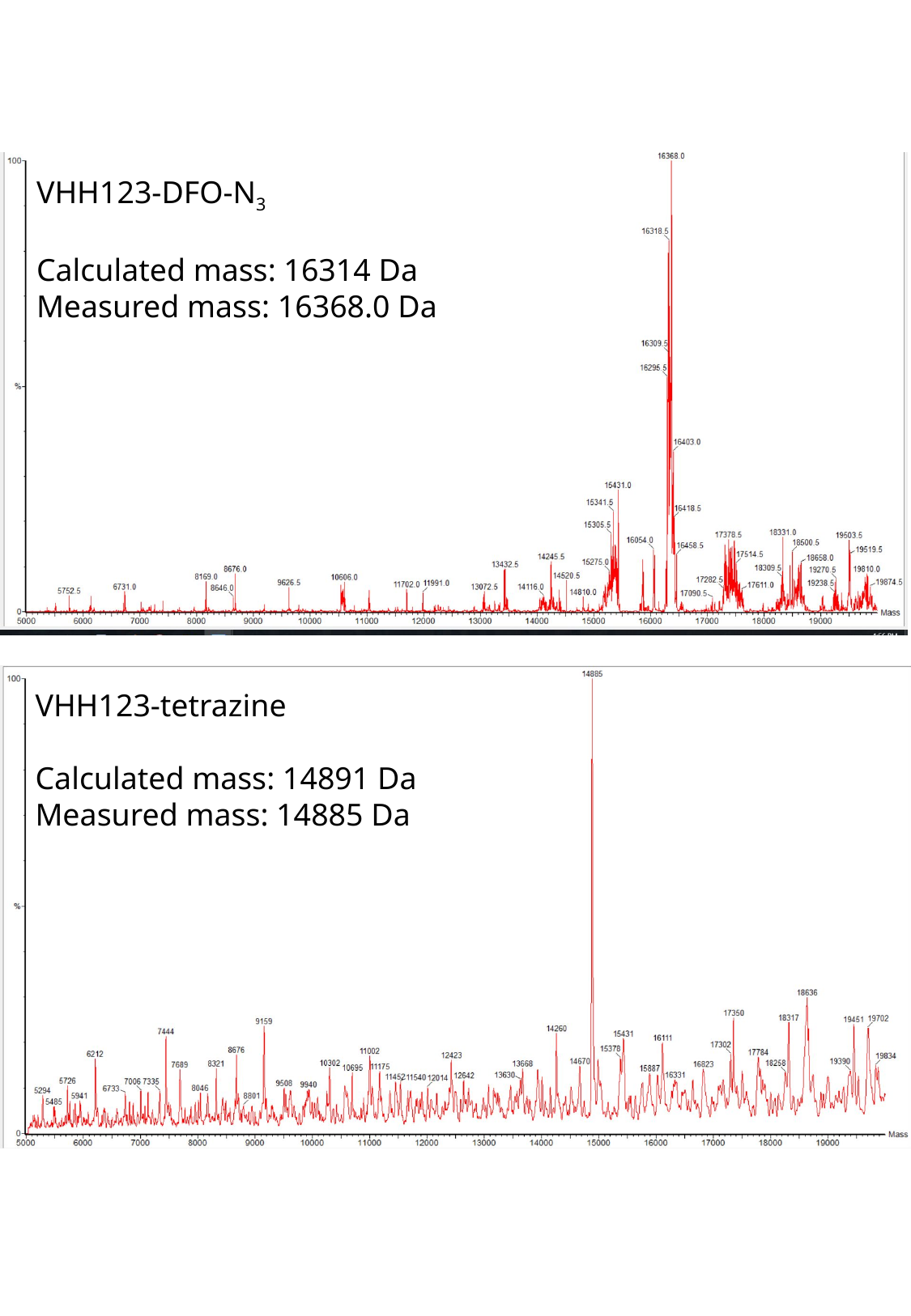

VHH123-DFO-N3
Calculated mass: 16314 Da
Measured mass: 16368.0 Da
VHH123-tetrazine
Calculated mass: 14891 Da
Measured mass: 14885 Da

## Slide 4
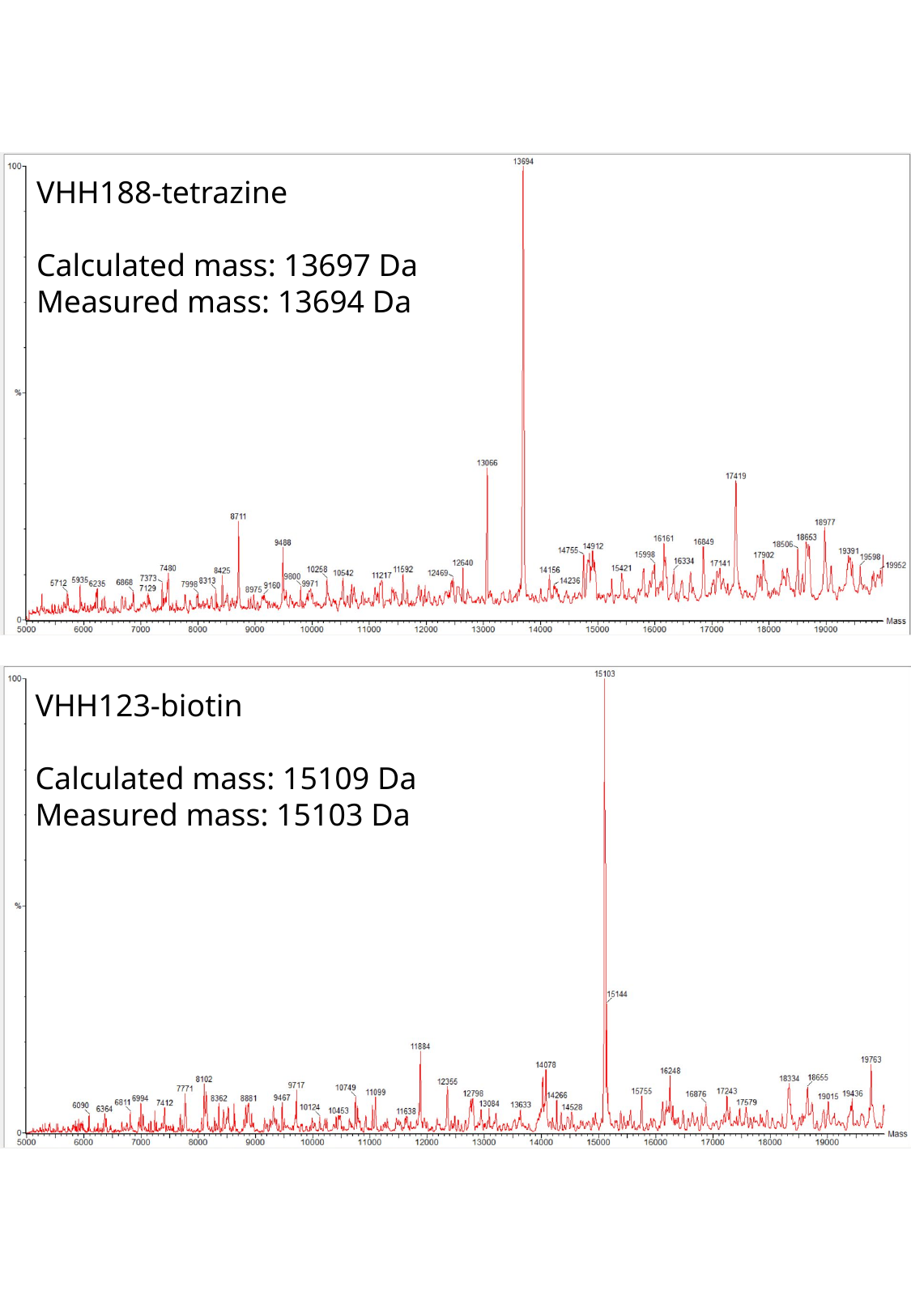

VHH188-tetrazine
Calculated mass: 13697 Da
Measured mass: 13694 Da
VHH123-biotin
Calculated mass: 15109 Da
Measured mass: 15103 Da

## Slide 5
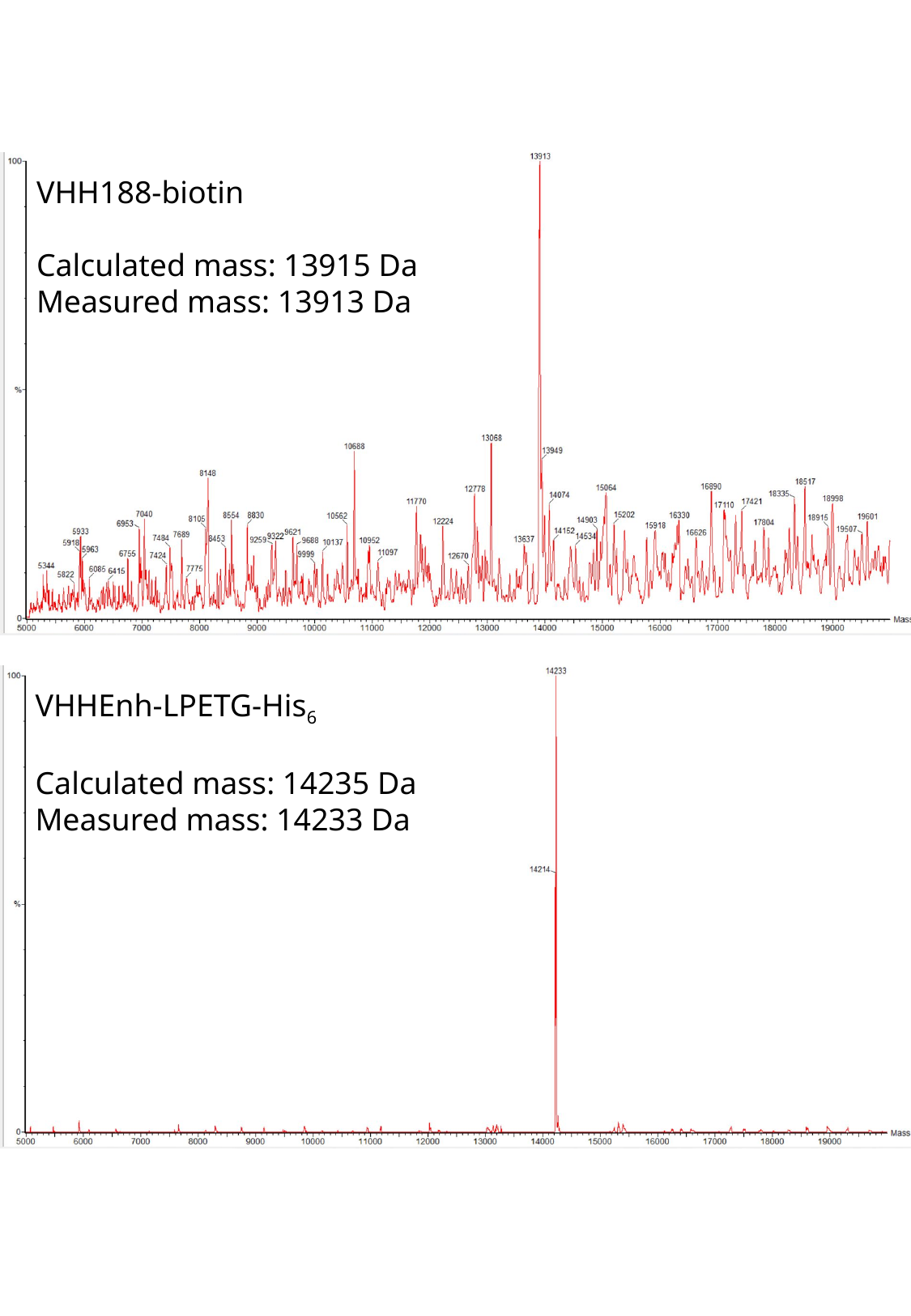

VHH188-biotin
Calculated mass: 13915 Da
Measured mass: 13913 Da
VHHEnh-LPETG-His6
Calculated mass: 14235 Da
Measured mass: 14233 Da

## Slide 6
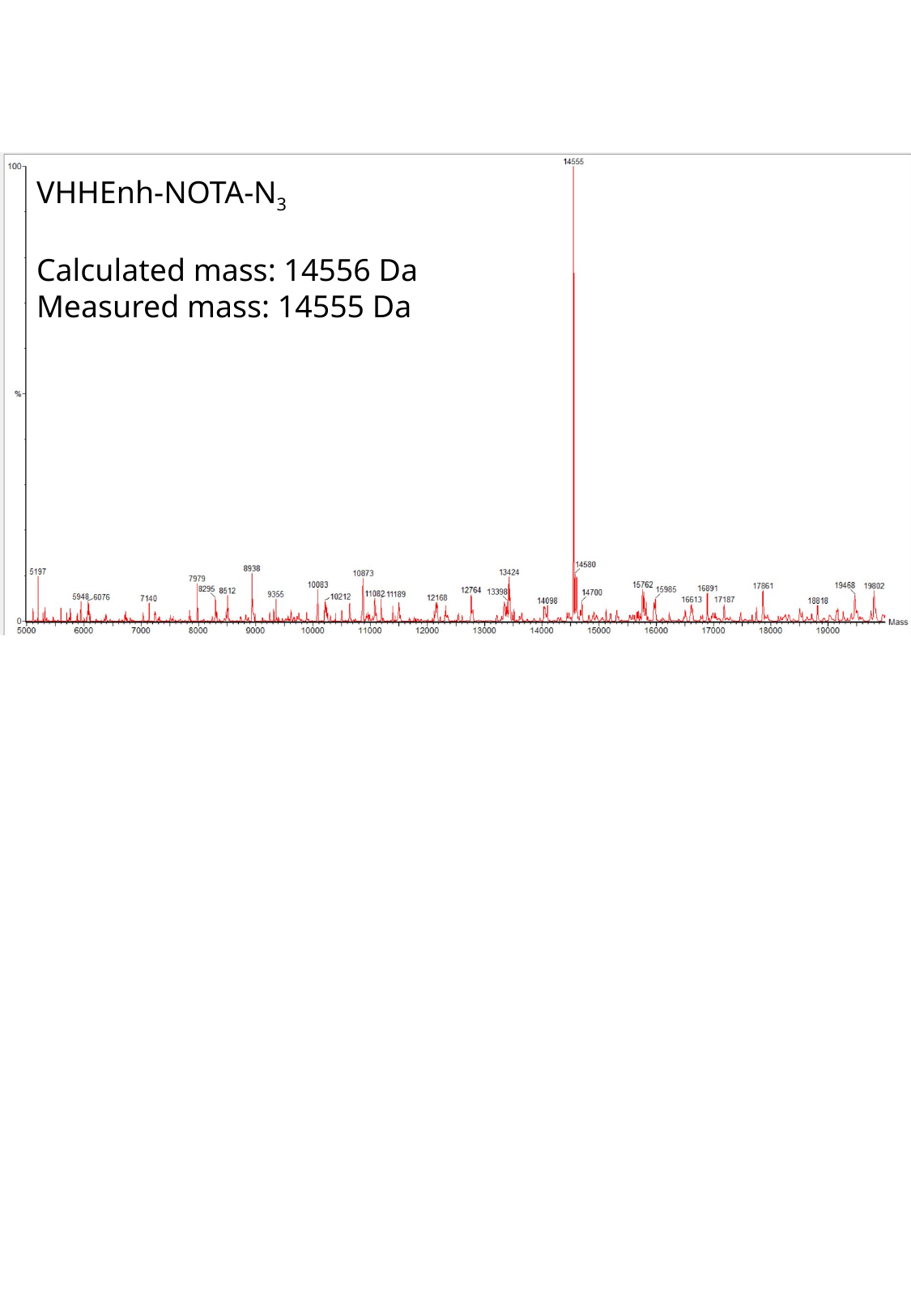

VHHEnh-NOTA-N3
Calculated mass: 14556 Da
Measured mass: 14555 Da
