# Supplementary material for: A pair of congenic mice for imaging of transplants by positron emission tomography using anti-transferrin receptor nanobodies
Source: eLife. 2025 Aug 18;14:RP104302. doi: 10.7554/eLife.104302 (PMC12360783; doi:10.7554/eLife.104302)
Supplement: Figure 4—source data 1. [file elife-104302-fig4-data1.zip › Figure 4-Source Data 1.pptx]

## Slide 1
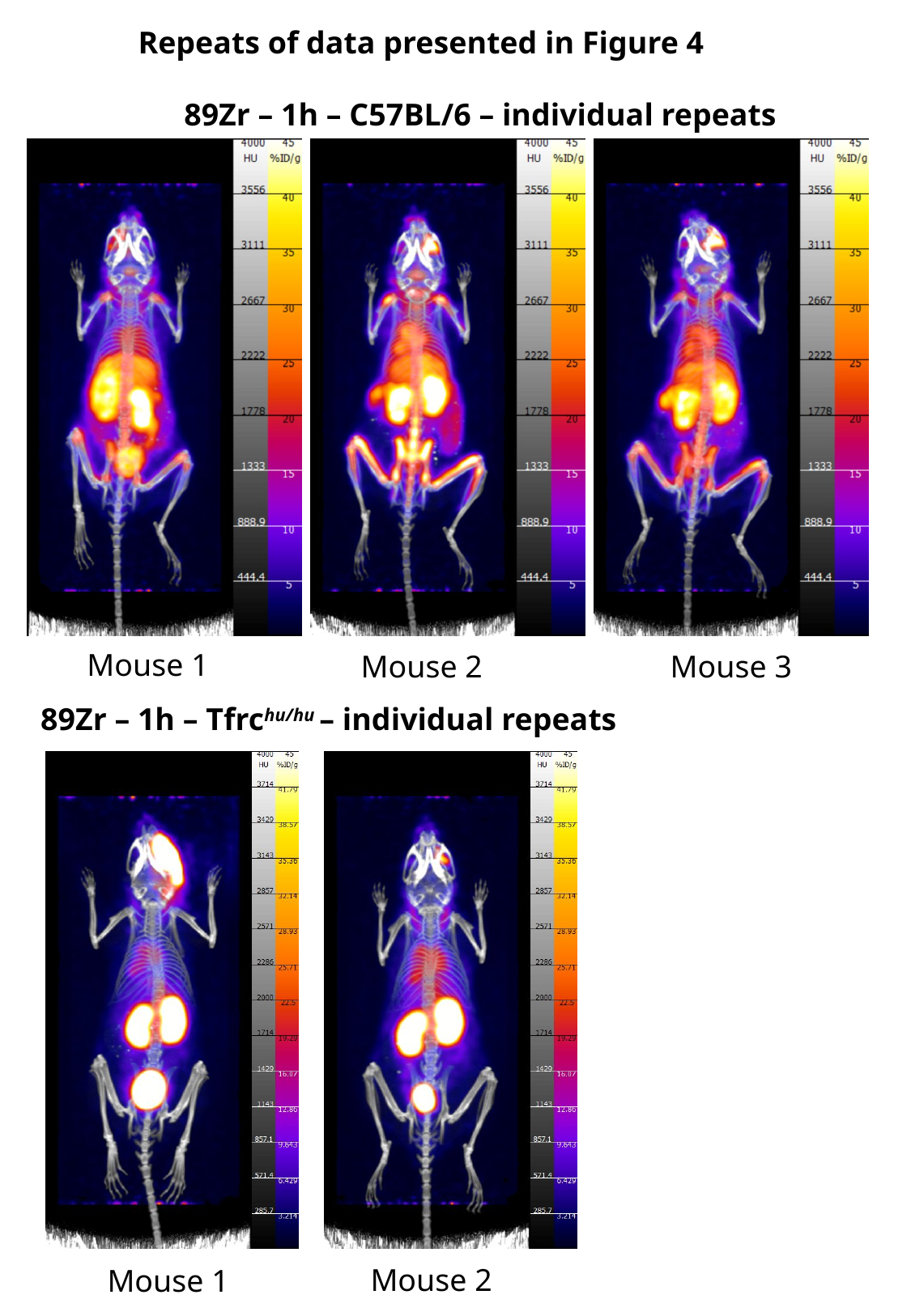

Repeats of data presented in Figure 4
89Zr – 1h – C57BL/6 – individual repeats
Mouse 1
Mouse 3
Mouse 2
89Zr – 1h – Tfrchu/hu – individual repeats
Mouse 2
Mouse 1

## Slide 2
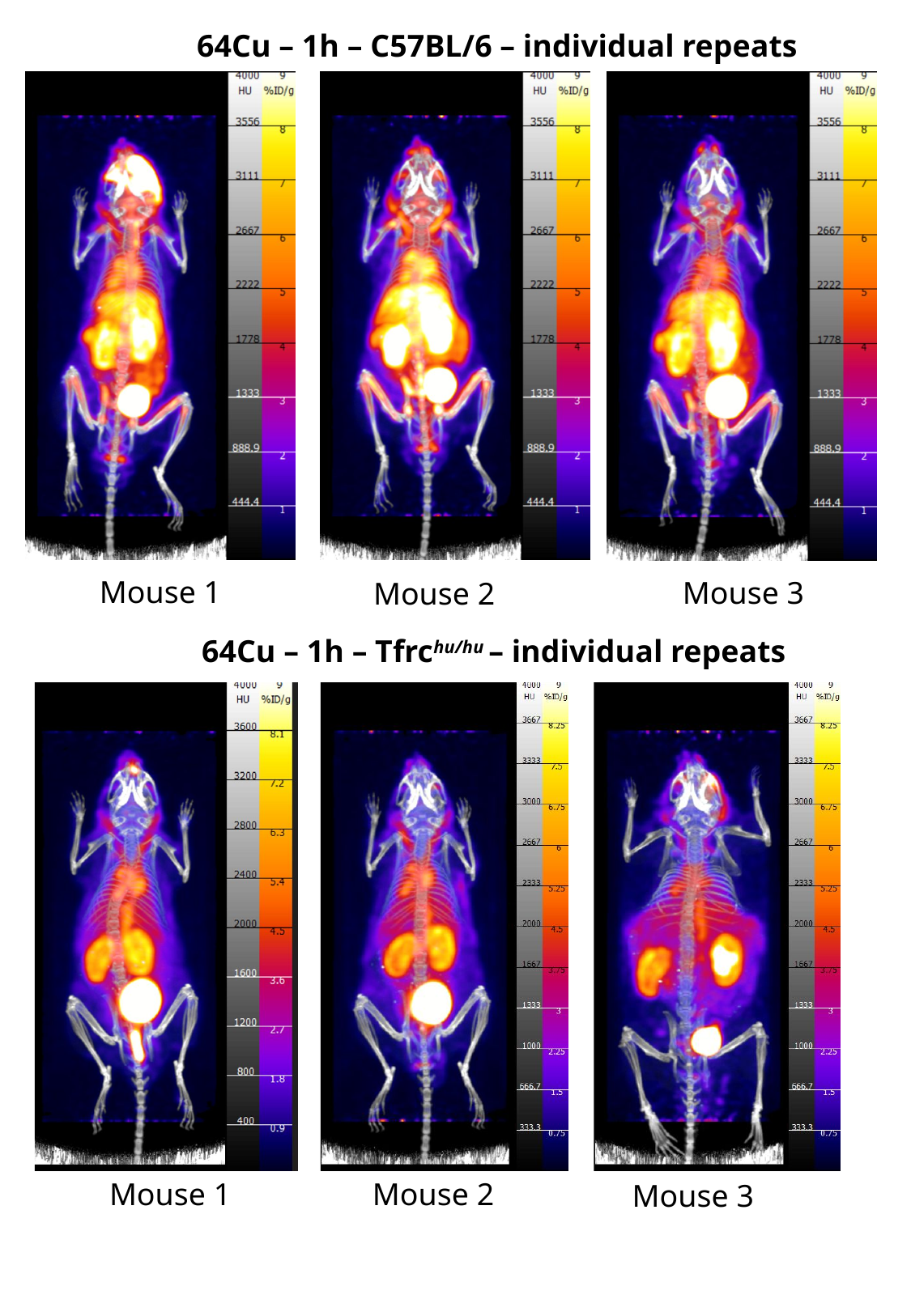

64Cu – 1h – C57BL/6 – individual repeats
Mouse 1
Mouse 3
Mouse 2
64Cu – 1h – Tfrchu/hu – individual repeats
Mouse 2
Mouse 1
Mouse 3

## Slide 3
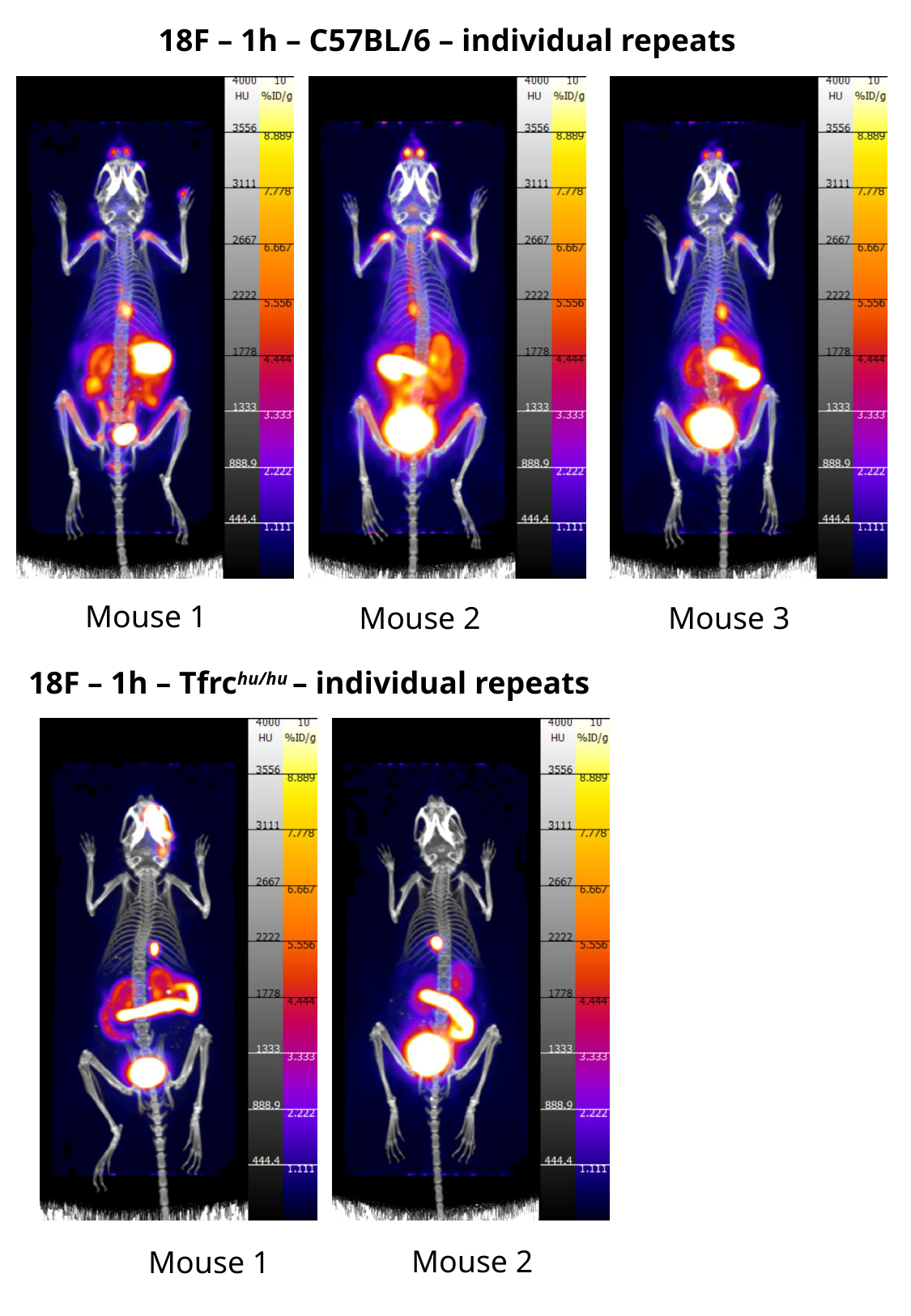

18F – 1h – C57BL/6 – individual repeats
Mouse 1
Mouse 3
Mouse 2
18F – 1h – Tfrchu/hu – individual repeats
Mouse 2
Mouse 1
