# Supplementary material for: A pair of congenic mice for imaging of transplants by positron emission tomography using anti-transferrin receptor nanobodies
Source: eLife. 2025 Aug 18;14:RP104302. doi: 10.7554/eLife.104302 (PMC12360783; doi:10.7554/eLife.104302)
Supplement: Figure 5—source data 1. [file elife-104302-fig5-data1.zip › Figure 5-Source Data 1.pptx]

## Slide 1
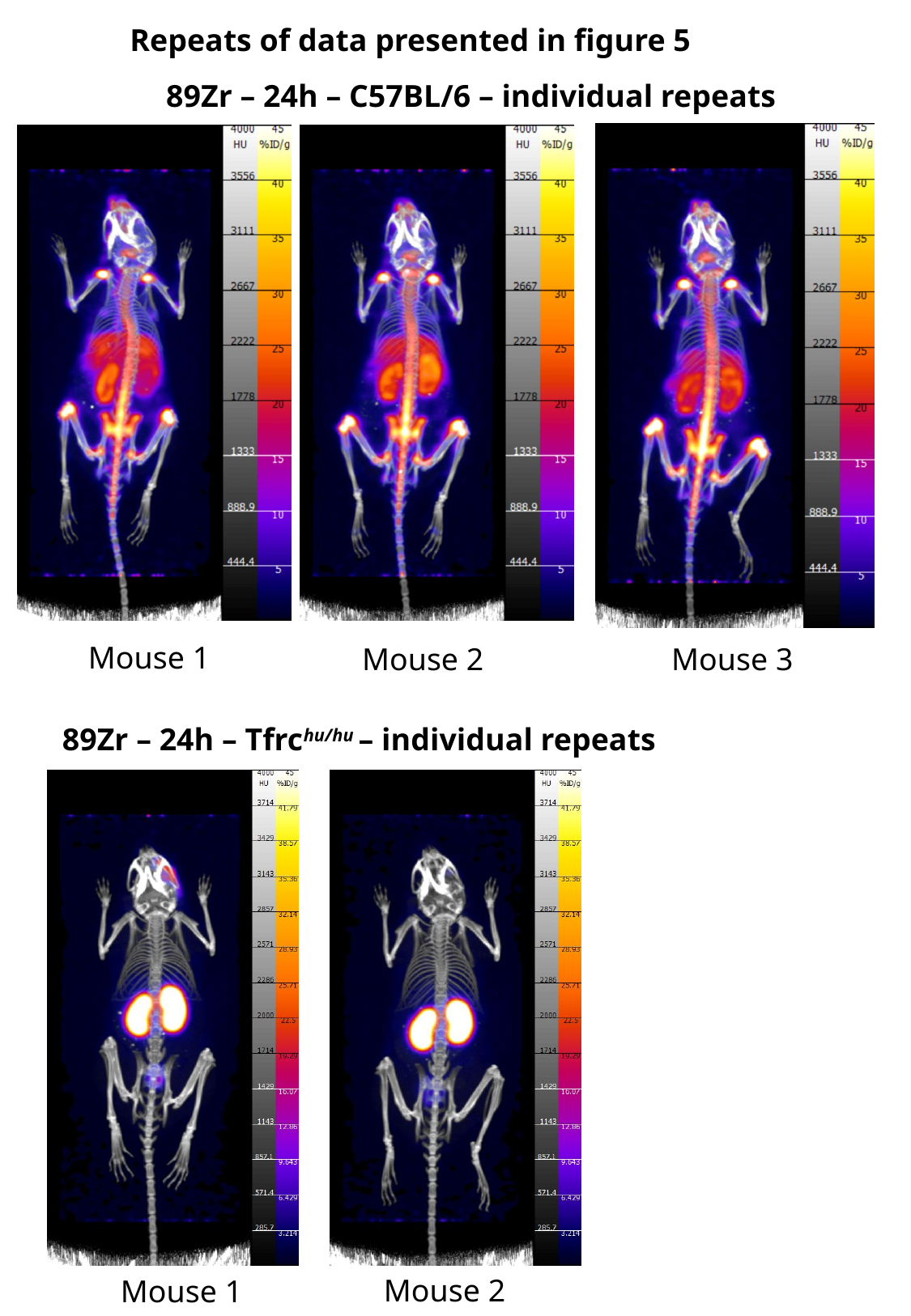

Repeats of data presented in figure 5
89Zr – 24h – C57BL/6 – individual repeats
Mouse 1
Mouse 3
Mouse 2
89Zr – 24h – Tfrchu/hu – individual repeats
Mouse 2
Mouse 1

## Slide 2
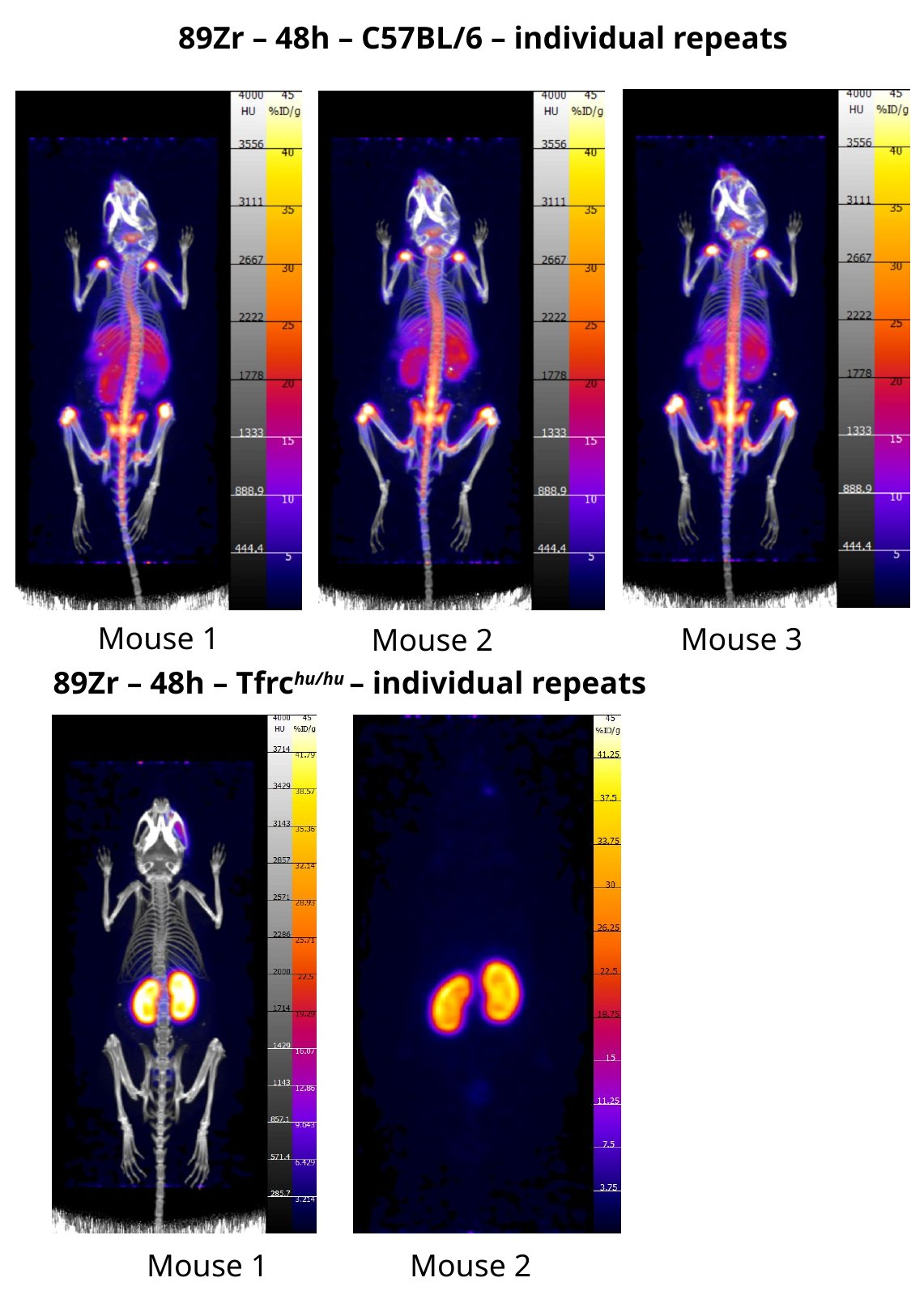

89Zr – 48h – C57BL/6 – individual repeats
Mouse 1
Mouse 3
Mouse 2
89Zr – 48h – Tfrchu/hu – individual repeats
Mouse 2
Mouse 1

## Slide 3
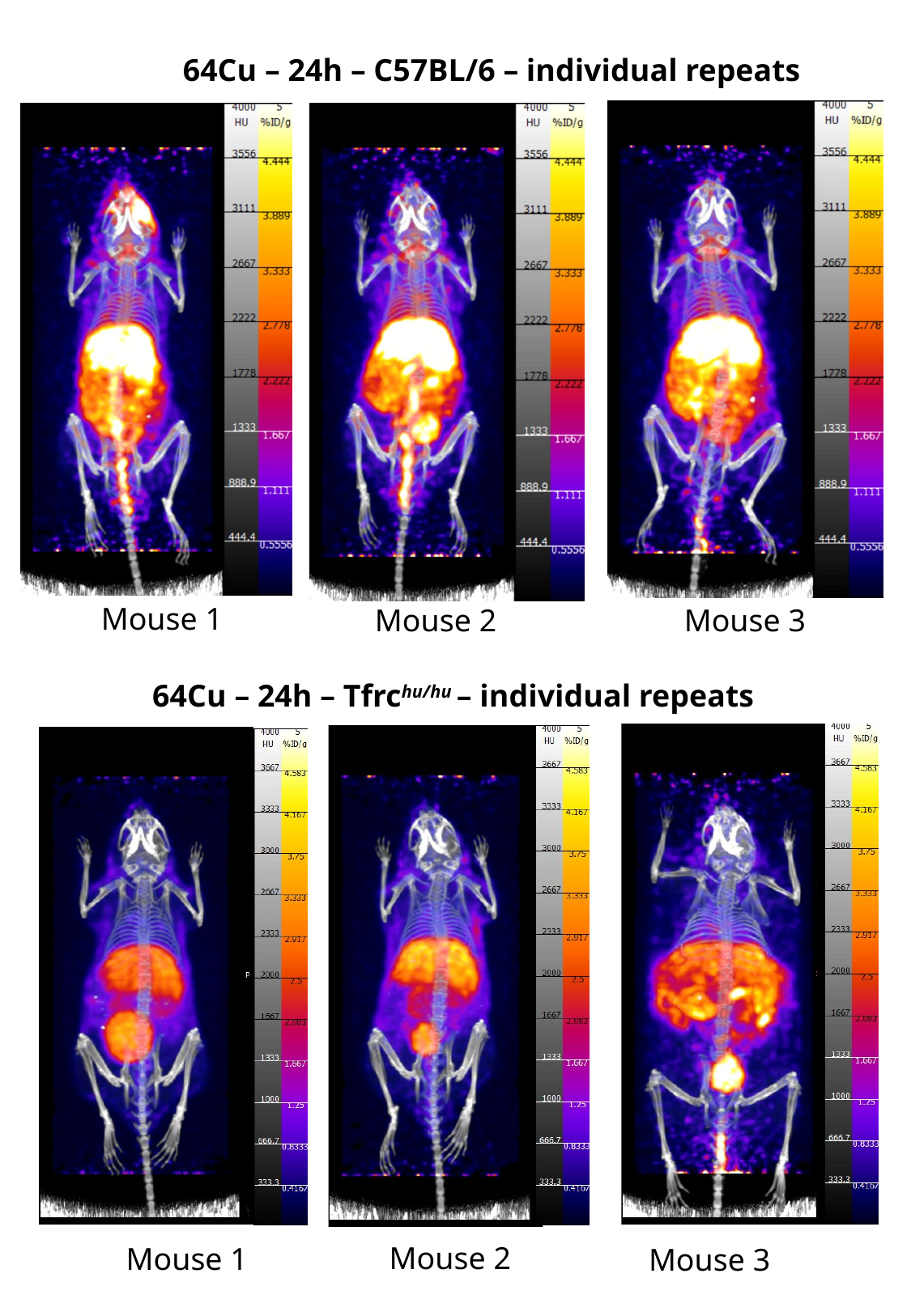

64Cu – 24h – C57BL/6 – individual repeats
Mouse 1
Mouse 3
Mouse 2
64Cu – 24h – Tfrchu/hu – individual repeats
Mouse 2
Mouse 1
Mouse 3
