# Supplementary material for: A pair of congenic mice for imaging of transplants by positron emission tomography using anti-transferrin receptor nanobodies
Source: eLife. 2025 Aug 18;14:RP104302. doi: 10.7554/eLife.104302 (PMC12360783; doi:10.7554/eLife.104302)
Supplement: Figure 6—source data 1. [file elife-104302-fig6-data1.zip › Figure 6-Source Data 1.pptx]

## Slide 1
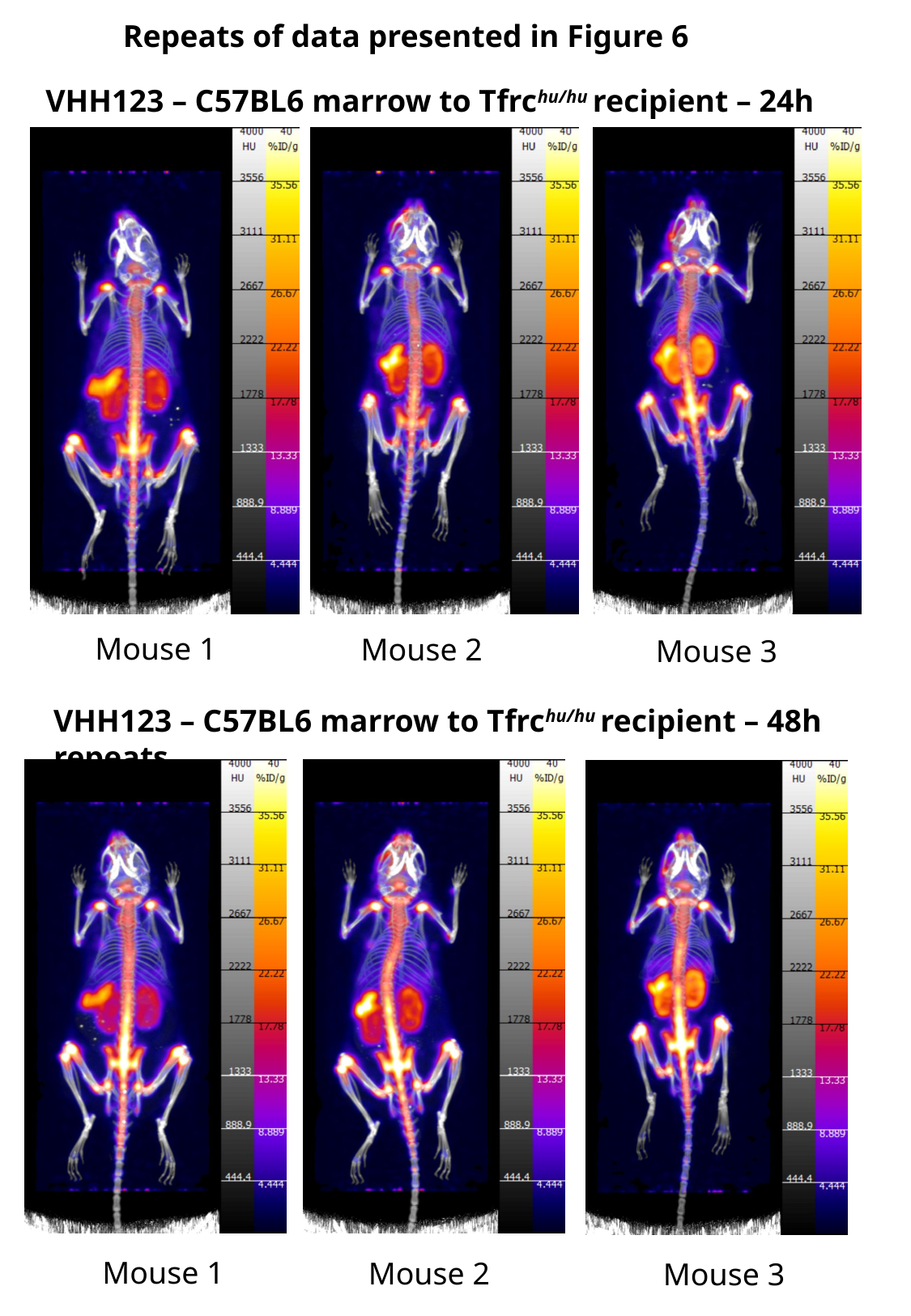

Repeats of data presented in Figure 6
VHH123 – C57BL6 marrow to Tfrchu/hu recipient – 24h repeats
Mouse 1
Mouse 2
Mouse 3
VHH123 – C57BL6 marrow to Tfrchu/hu recipient – 48h repeats
Mouse 1
Mouse 2
Mouse 3

## Slide 2
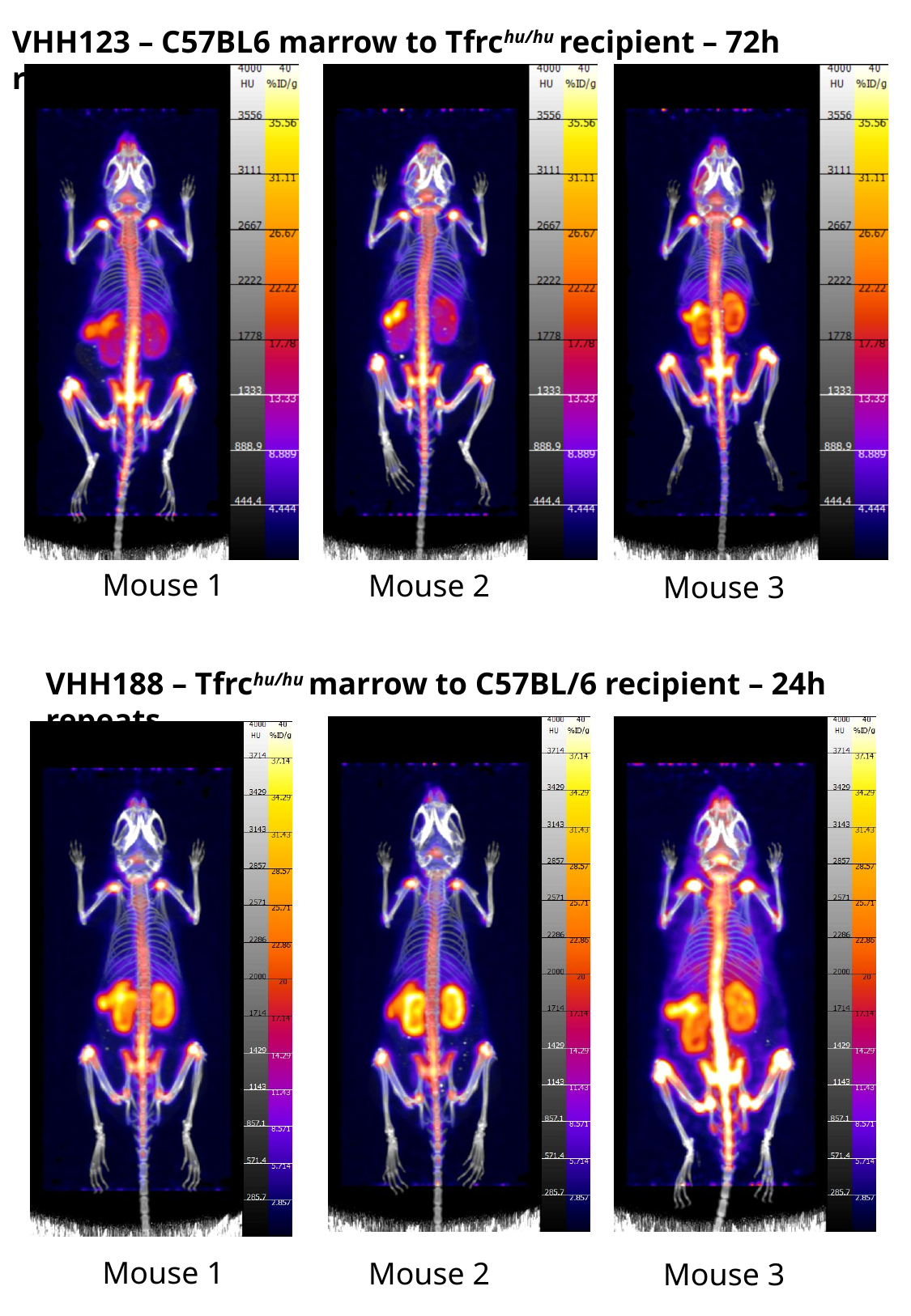

VHH123 – C57BL6 marrow to Tfrchu/hu recipient – 72h repeats
Mouse 1
Mouse 2
Mouse 3
VHH188 – Tfrchu/hu marrow to C57BL/6 recipient – 24h repeats
Mouse 1
Mouse 2
Mouse 3

## Slide 3
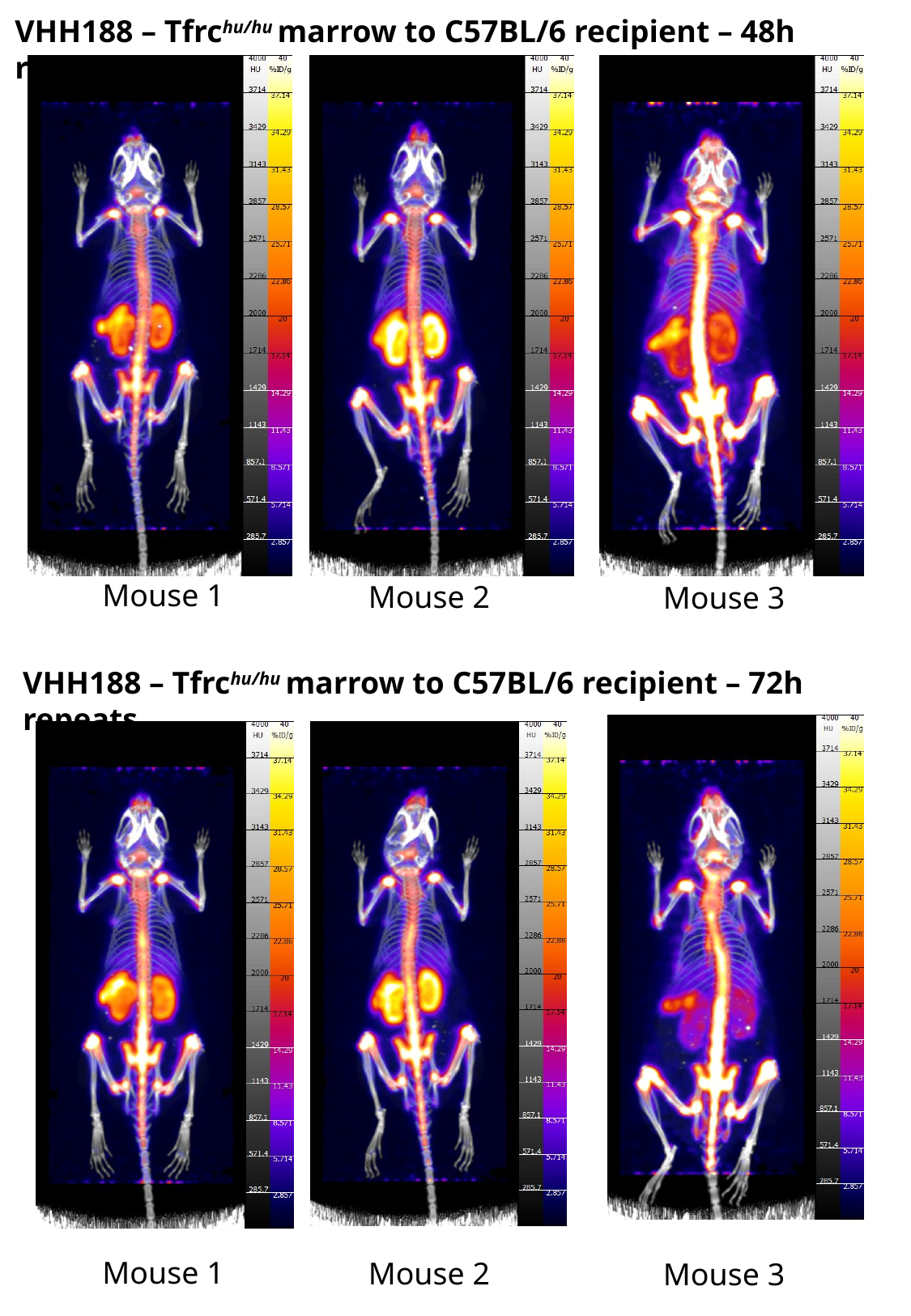

VHH188 – Tfrchu/hu marrow to C57BL/6 recipient – 48h repeats
Mouse 1
Mouse 2
Mouse 3
VHH188 – Tfrchu/hu marrow to C57BL/6 recipient – 72h repeats
Mouse 1
Mouse 2
Mouse 3

## Slide 4
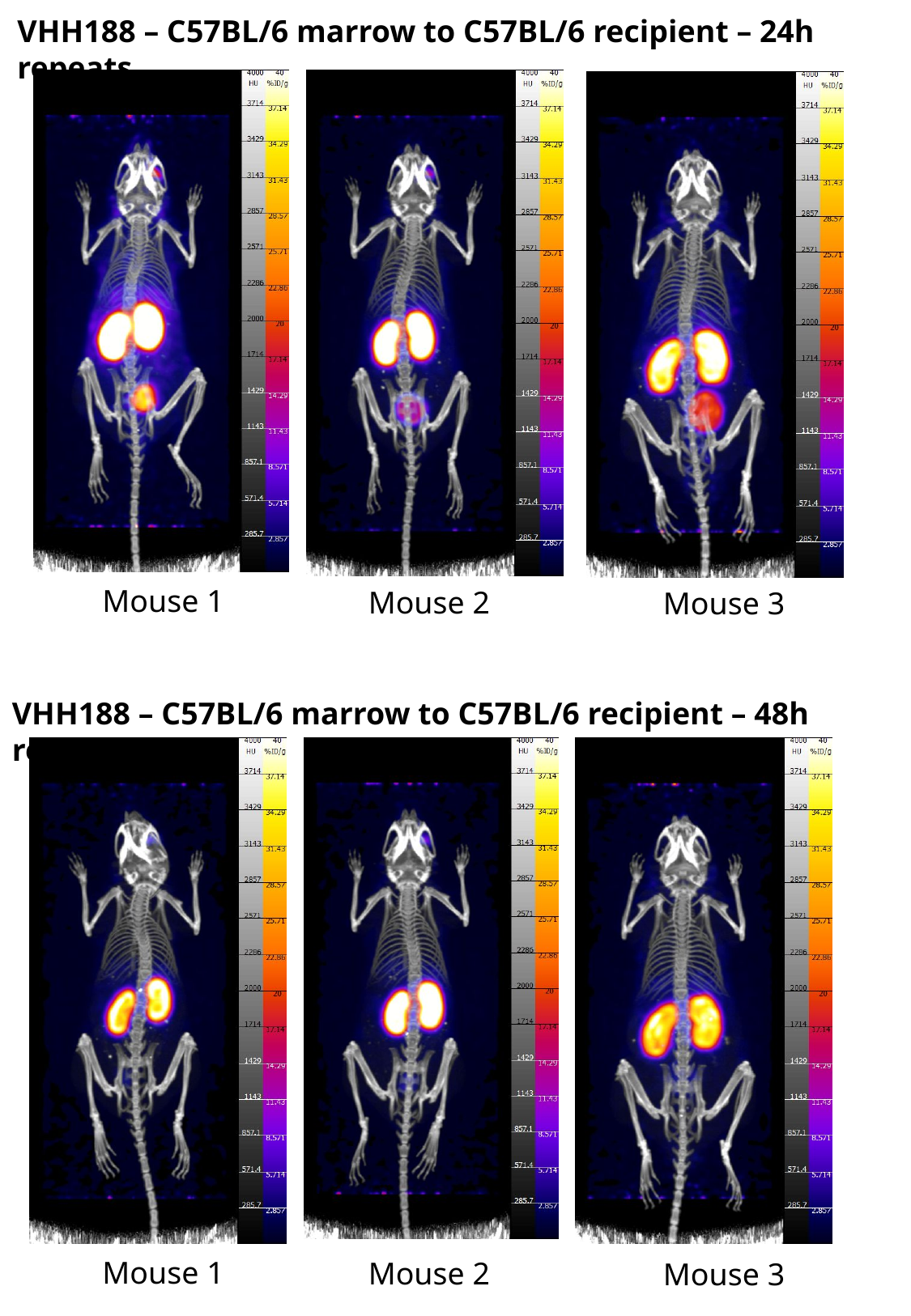

VHH188 – C57BL/6 marrow to C57BL/6 recipient – 24h repeats
Mouse 1
Mouse 2
Mouse 3
VHH188 – C57BL/6 marrow to C57BL/6 recipient – 48h repeats
Mouse 1
Mouse 2
Mouse 3

## Slide 5
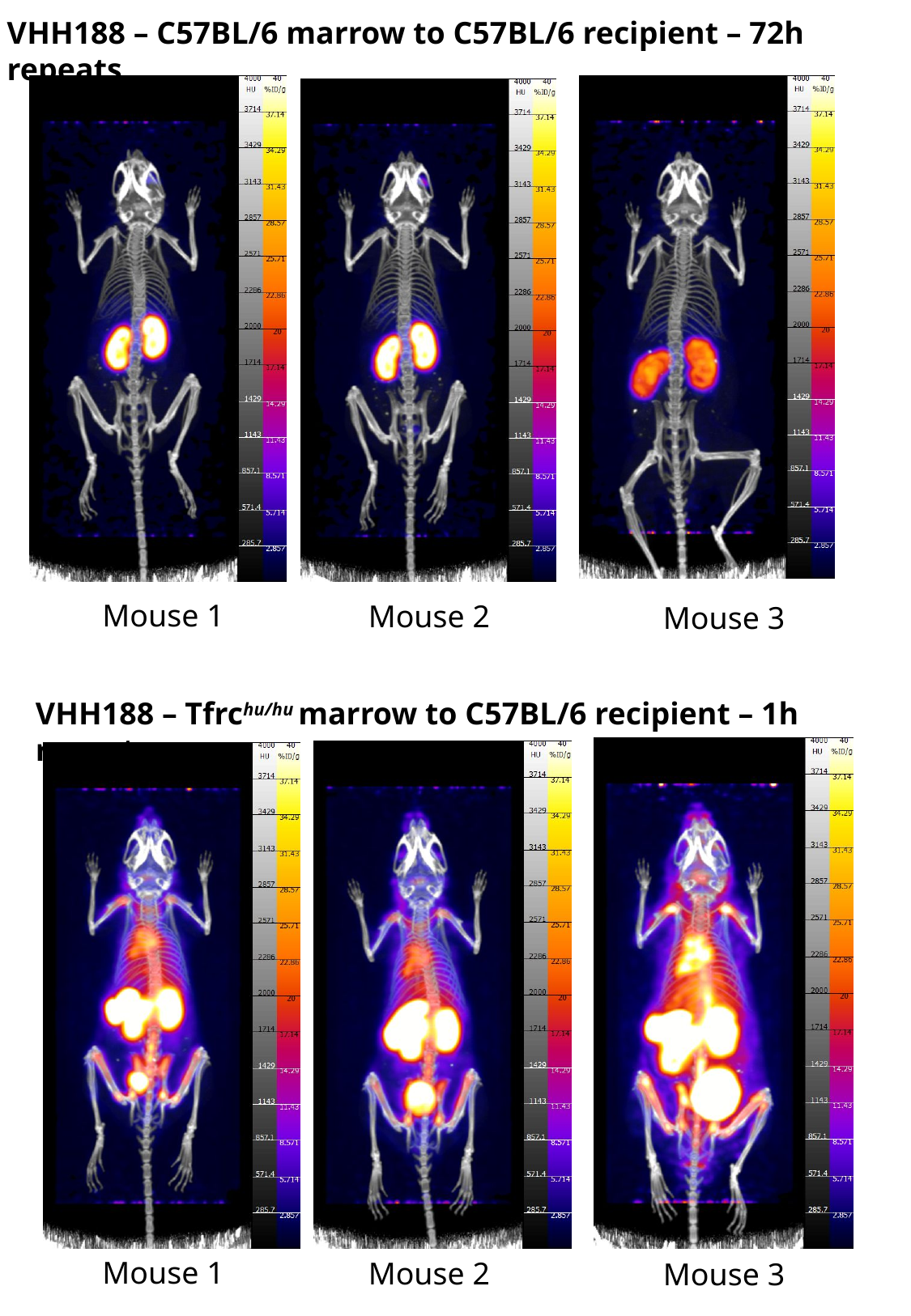

VHH188 – C57BL/6 marrow to C57BL/6 recipient – 72h repeats
Mouse 1
Mouse 2
Mouse 3
VHH188 – Tfrchu/hu marrow to C57BL/6 recipient – 1h repeats
Mouse 1
Mouse 2
Mouse 3

## Slide 6
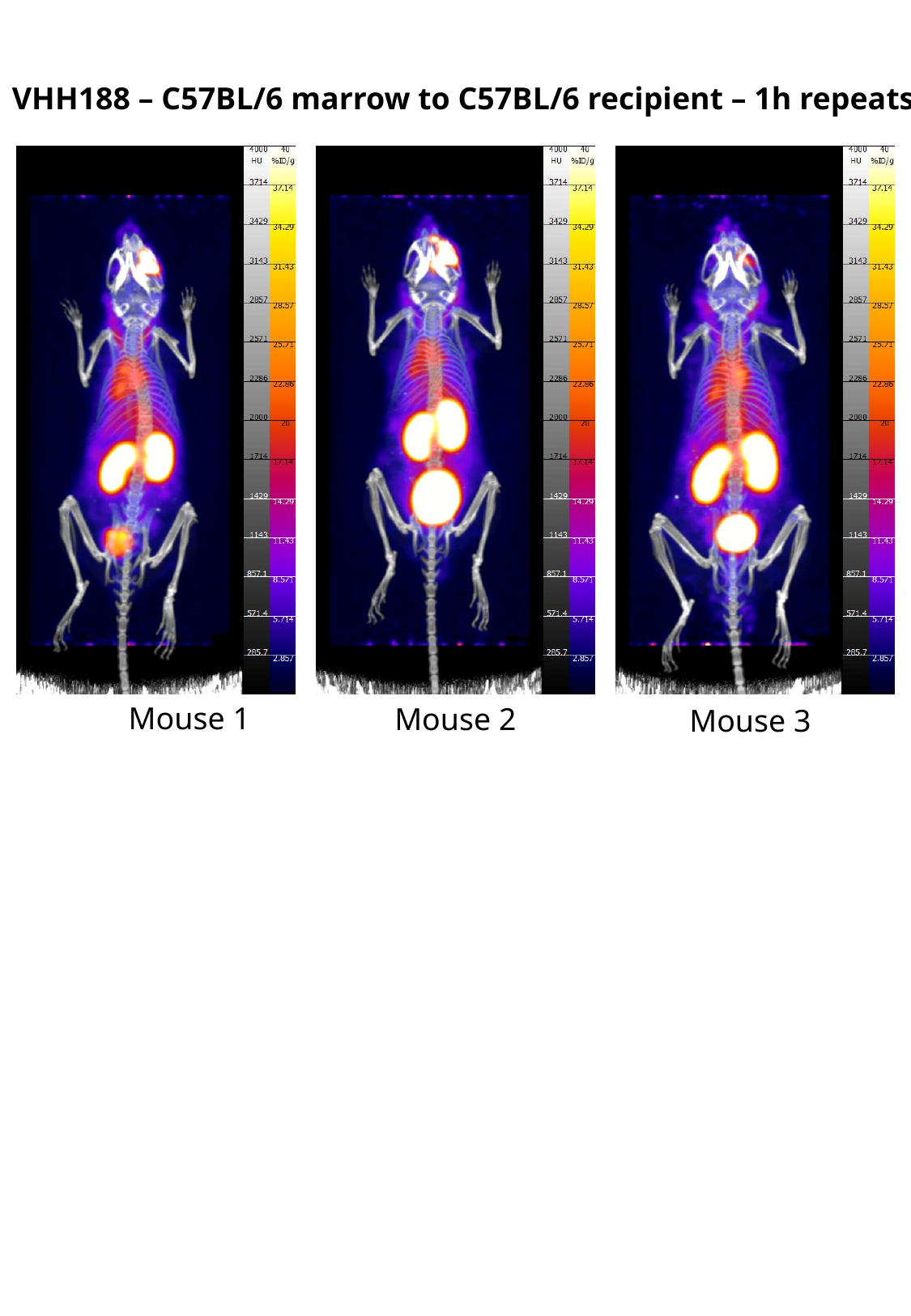

VHH188 – C57BL/6 marrow to C57BL/6 recipient – 1h repeats
Mouse 1
Mouse 2
Mouse 3
