# Supplementary material for: A pair of congenic mice for imaging of transplants by positron emission tomography using anti-transferrin receptor nanobodies
Source: eLife. 2025 Aug 18;14:RP104302. doi: 10.7554/eLife.104302 (PMC12360783; doi:10.7554/eLife.104302)
Supplement: Figure 7—source data 1. [file elife-104302-fig7-data1.zip › Figure 7-Source Data 1.pptx]

## Slide 1
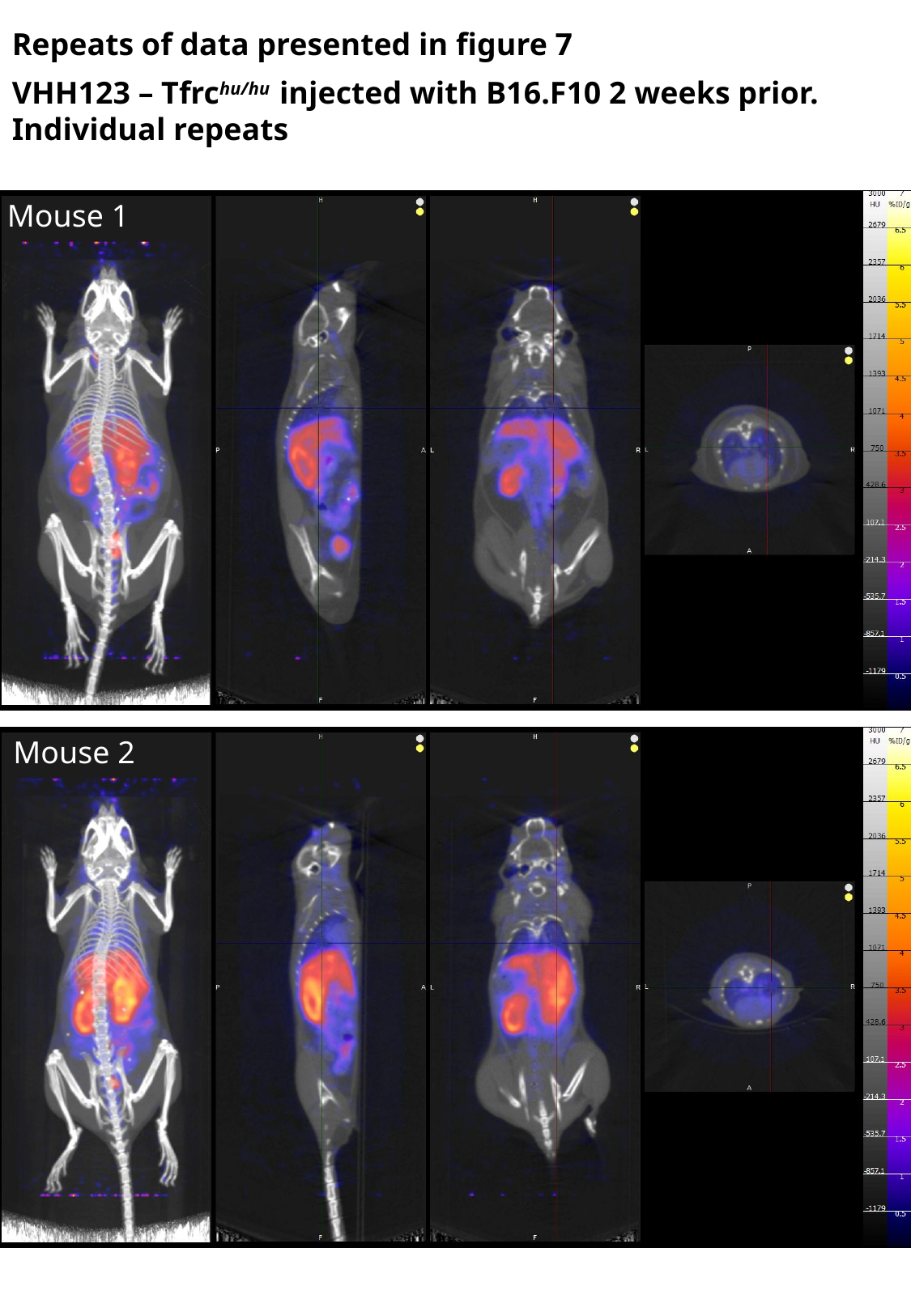

Repeats of data presented in figure 7
VHH123 – Tfrchu/hu injected with B16.F10 2 weeks prior.
Individual repeats
Mouse 1
Mouse 2

## Slide 2
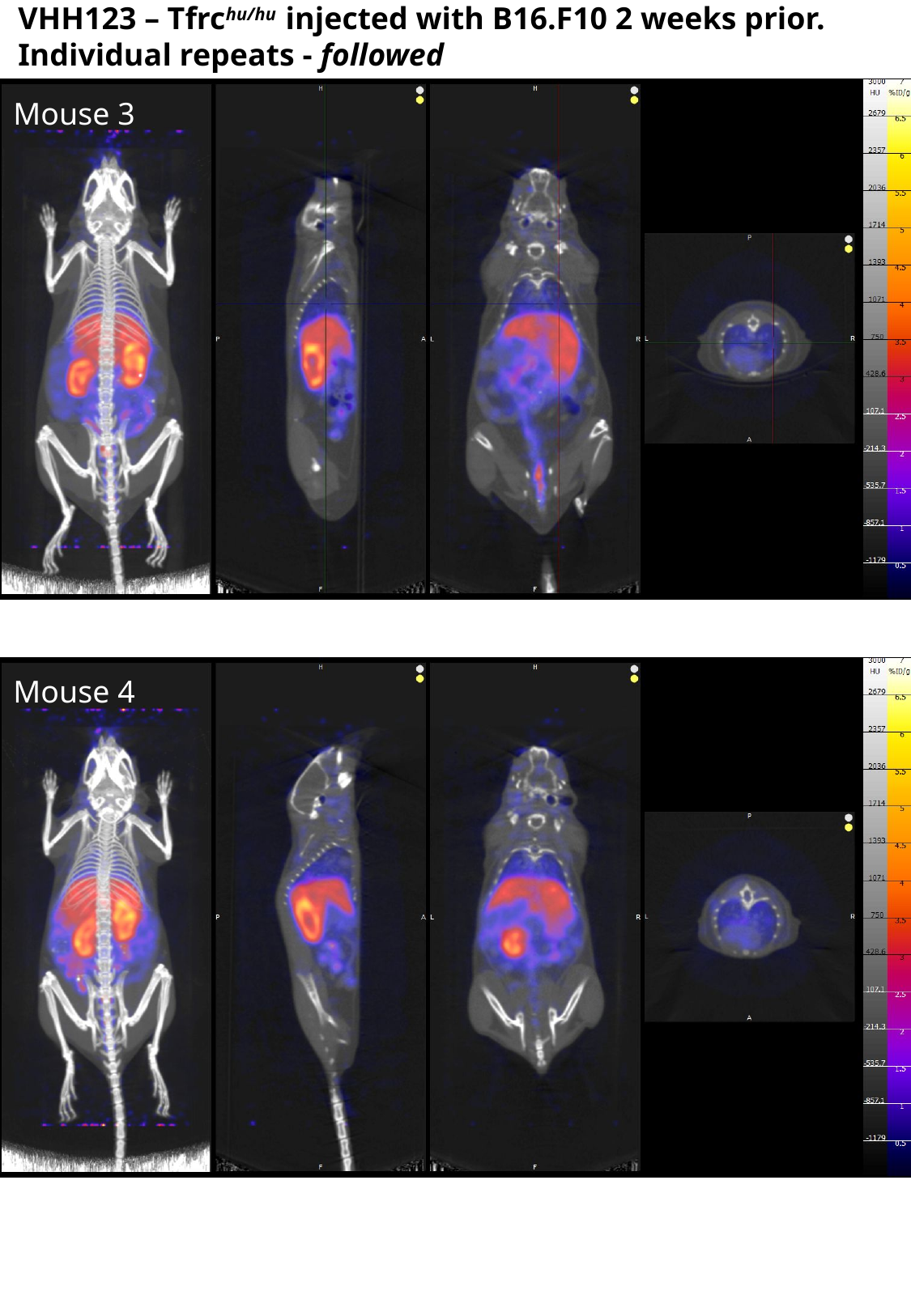

VHH123 – Tfrchu/hu injected with B16.F10 2 weeks prior.
Individual repeats - followed
Mouse 3
Mouse 4

## Slide 3
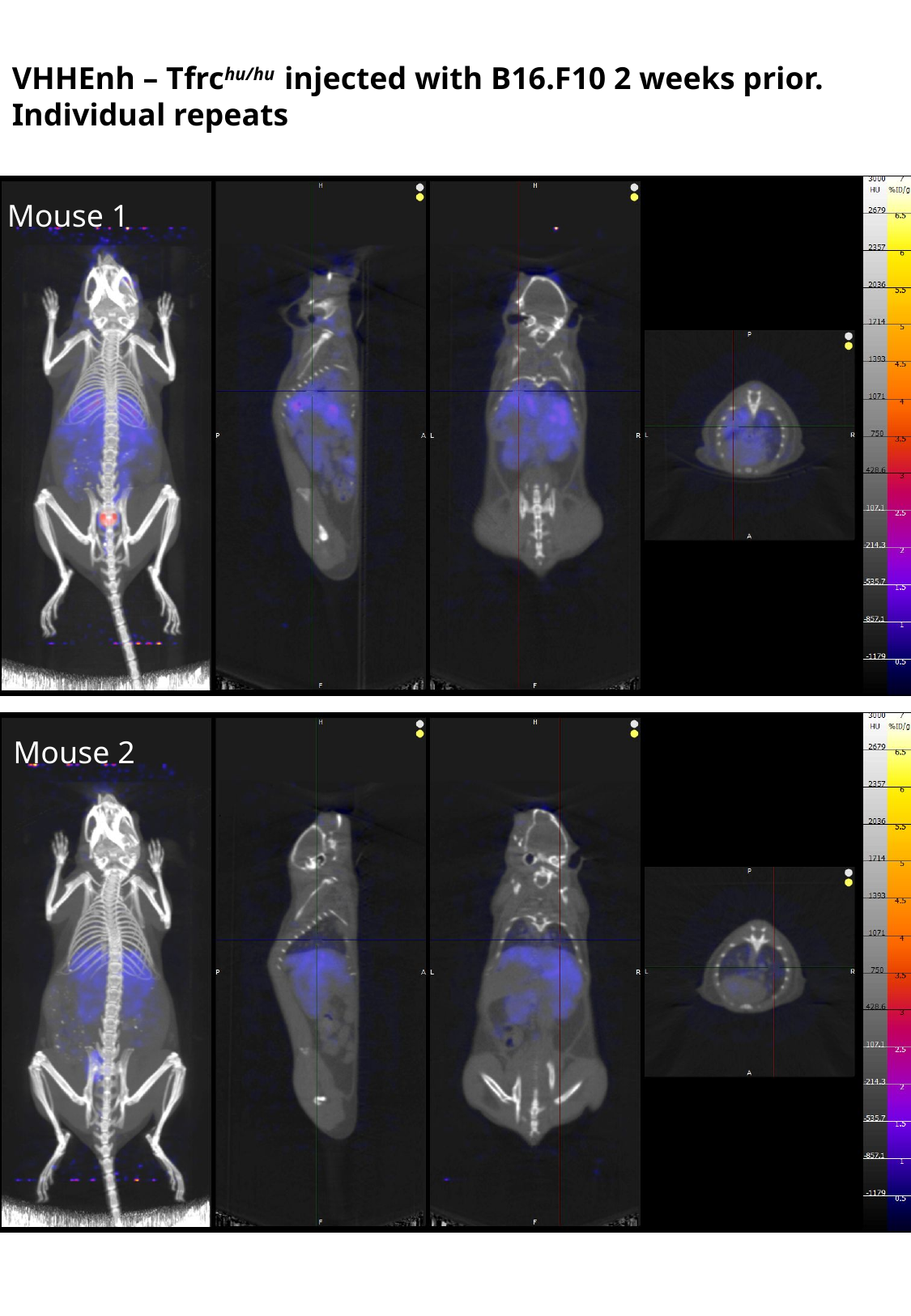

VHHEnh – Tfrchu/hu injected with B16.F10 2 weeks prior.
Individual repeats
Mouse 1
Mouse 2

## Slide 4
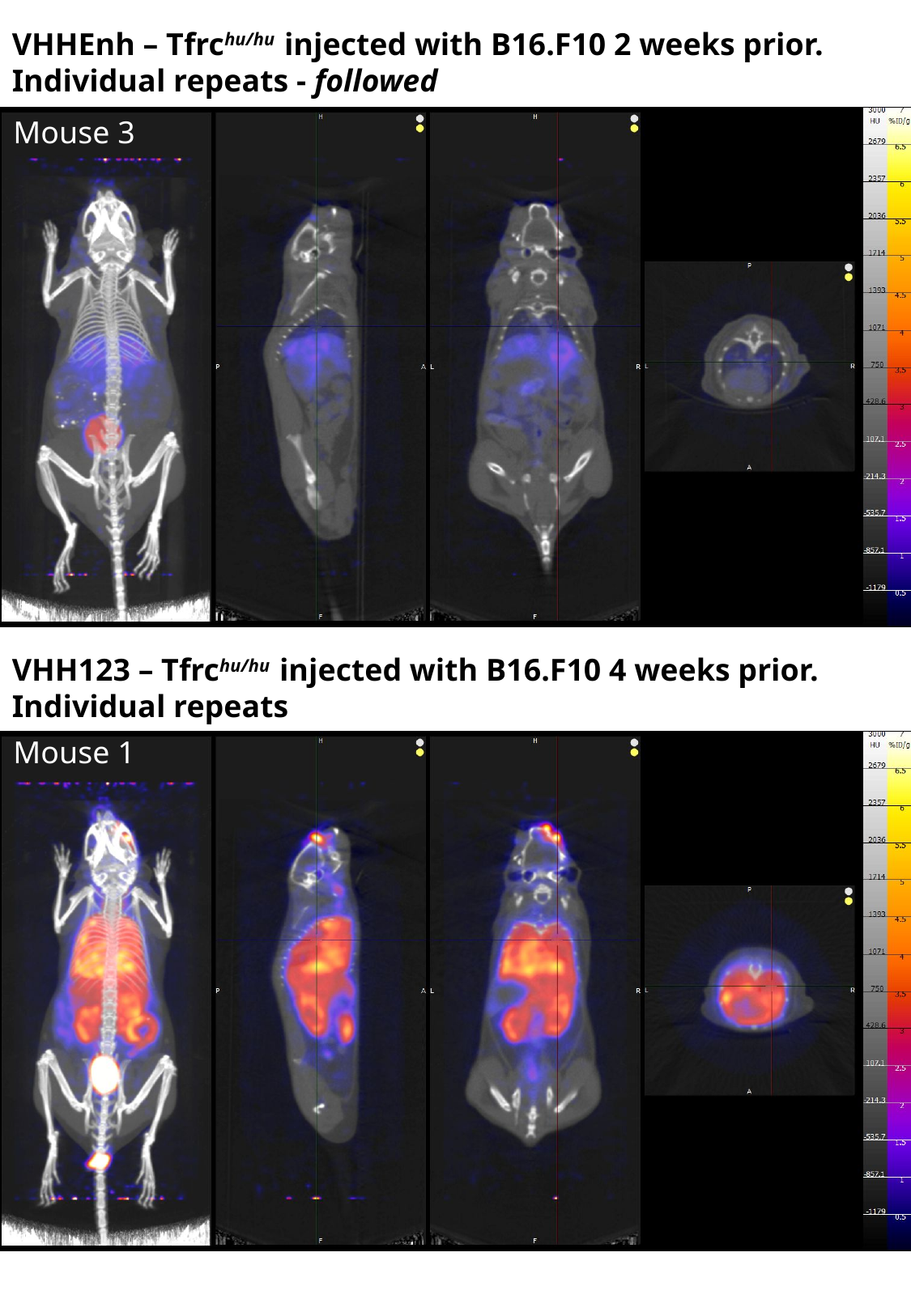

VHHEnh – Tfrchu/hu injected with B16.F10 2 weeks prior.
Individual repeats - followed
Mouse 3
VHH123 – Tfrchu/hu injected with B16.F10 4 weeks prior.
Individual repeats
Mouse 1

## Slide 5
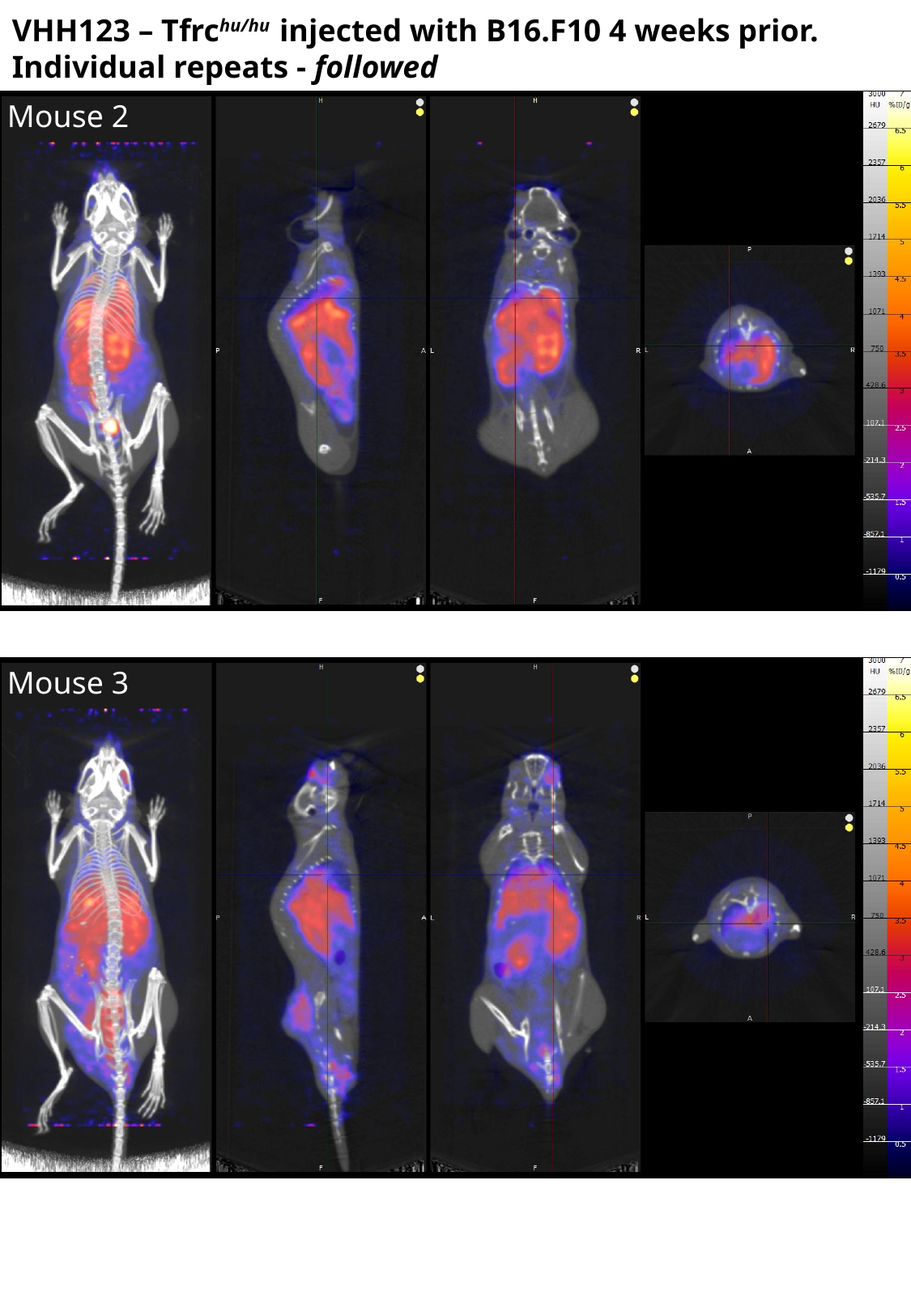

VHH123 – Tfrchu/hu injected with B16.F10 4 weeks prior.
Individual repeats - followed
Mouse 2
Mouse 3

## Slide 6
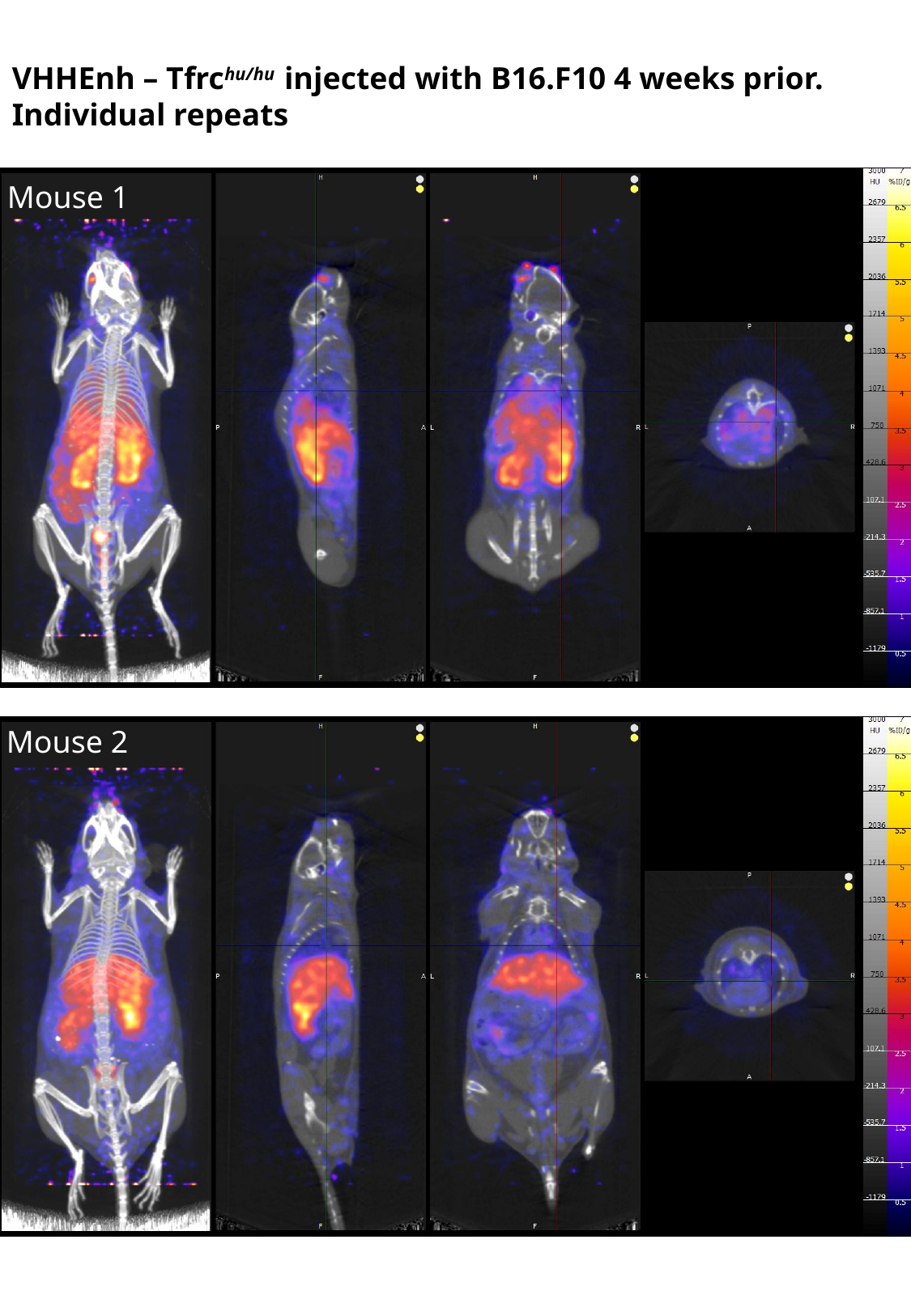

VHHEnh – Tfrchu/hu injected with B16.F10 4 weeks prior.
Individual repeats
Mouse 1
Mouse 2

## Slide 7
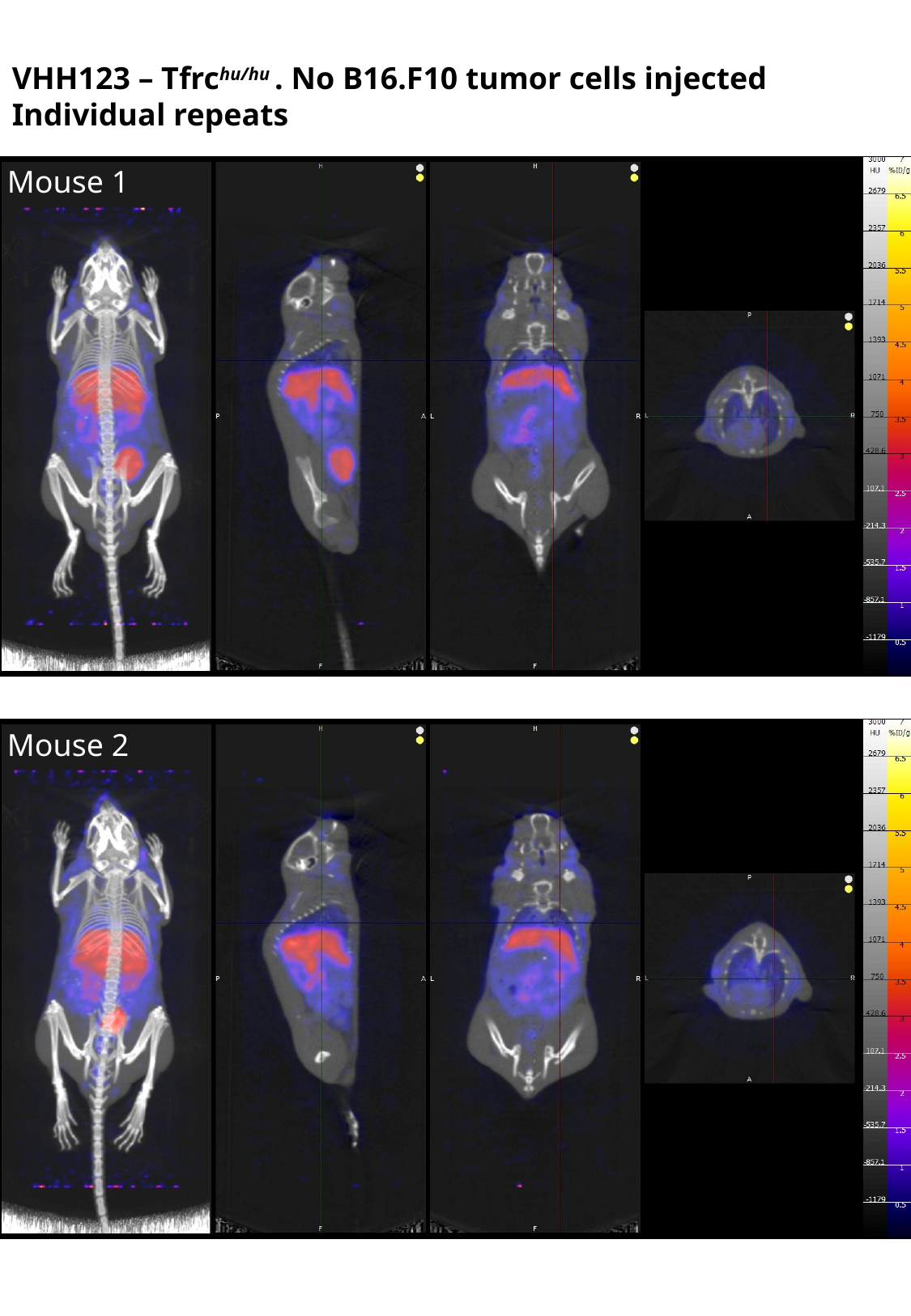

VHH123 – Tfrchu/hu . No B16.F10 tumor cells injected
Individual repeats
Mouse 1
Mouse 2

## Slide 8
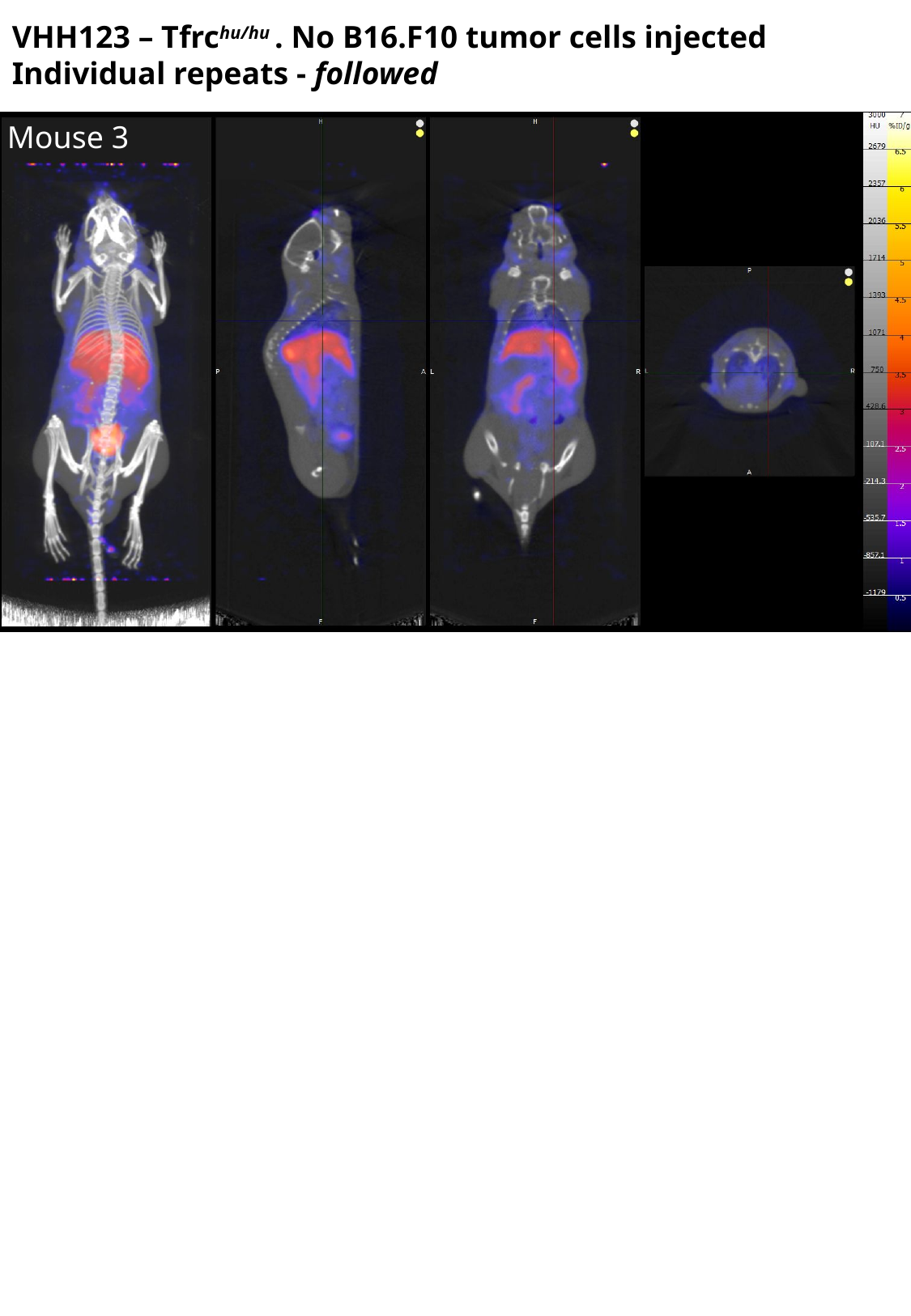

VHH123 – Tfrchu/hu . No B16.F10 tumor cells injected
Individual repeats - followed
Mouse 3
