# Supplementary material for: A pair of congenic mice for imaging of transplants by positron emission tomography using anti-transferrin receptor nanobodies
Source: eLife. 2025 Aug 18;14:RP104302. doi: 10.7554/eLife.104302 (PMC12360783; doi:10.7554/eLife.104302)
Supplement: Figure 8—source data 1. [file elife-104302-fig8-data1.zip › Figure 8-Source Data 1.pptx]

## Slide 1
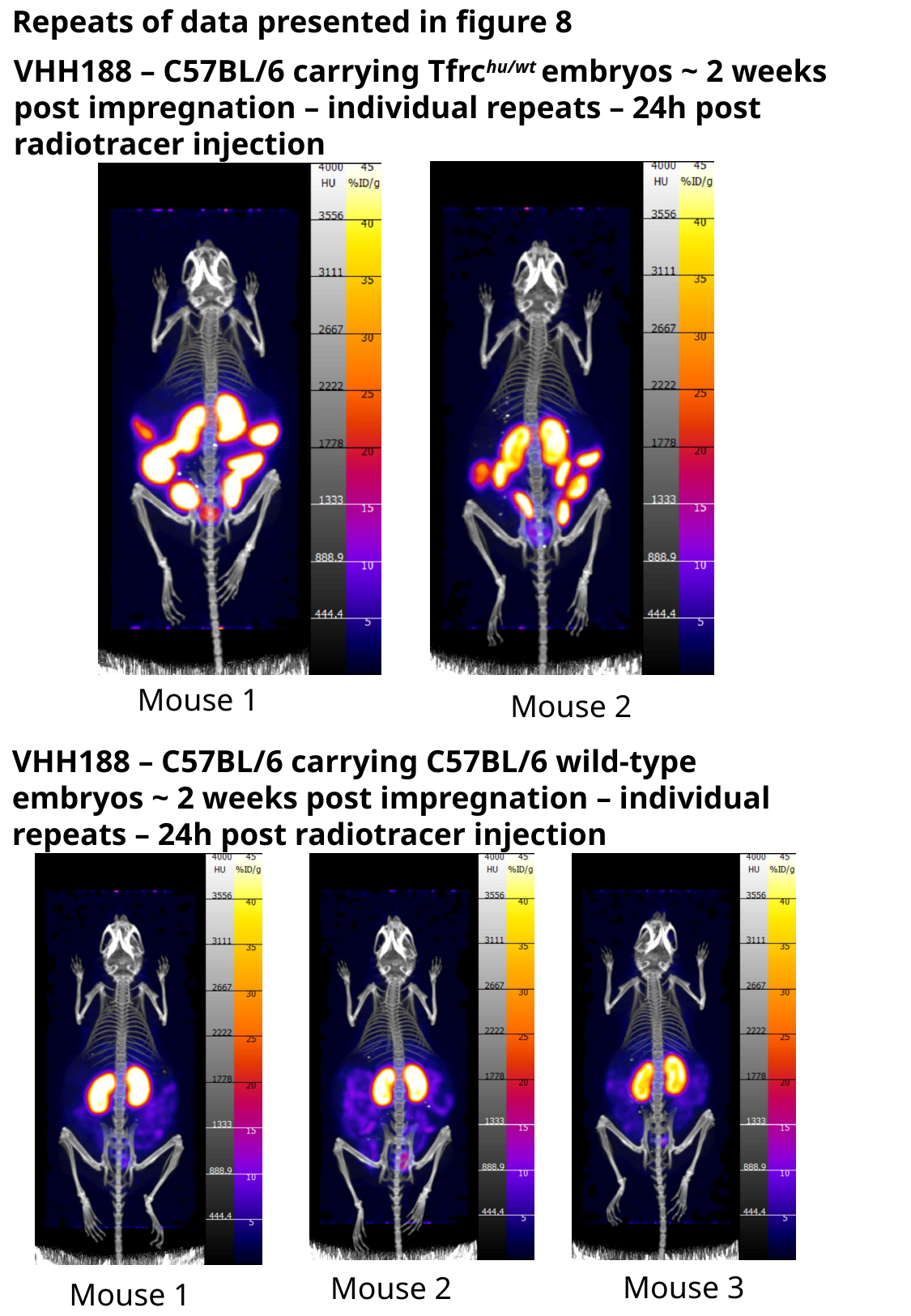

Repeats of data presented in figure 8
VHH188 – C57BL/6 carrying Tfrchu/wt embryos ~ 2 weeks post impregnation – individual repeats – 24h post radiotracer injection
Mouse 1
Mouse 2
VHH188 – C57BL/6 carrying C57BL/6 wild-type embryos ~ 2 weeks post impregnation – individual repeats – 24h post radiotracer injection
Mouse 3
Mouse 2
Mouse 1

## Slide 2
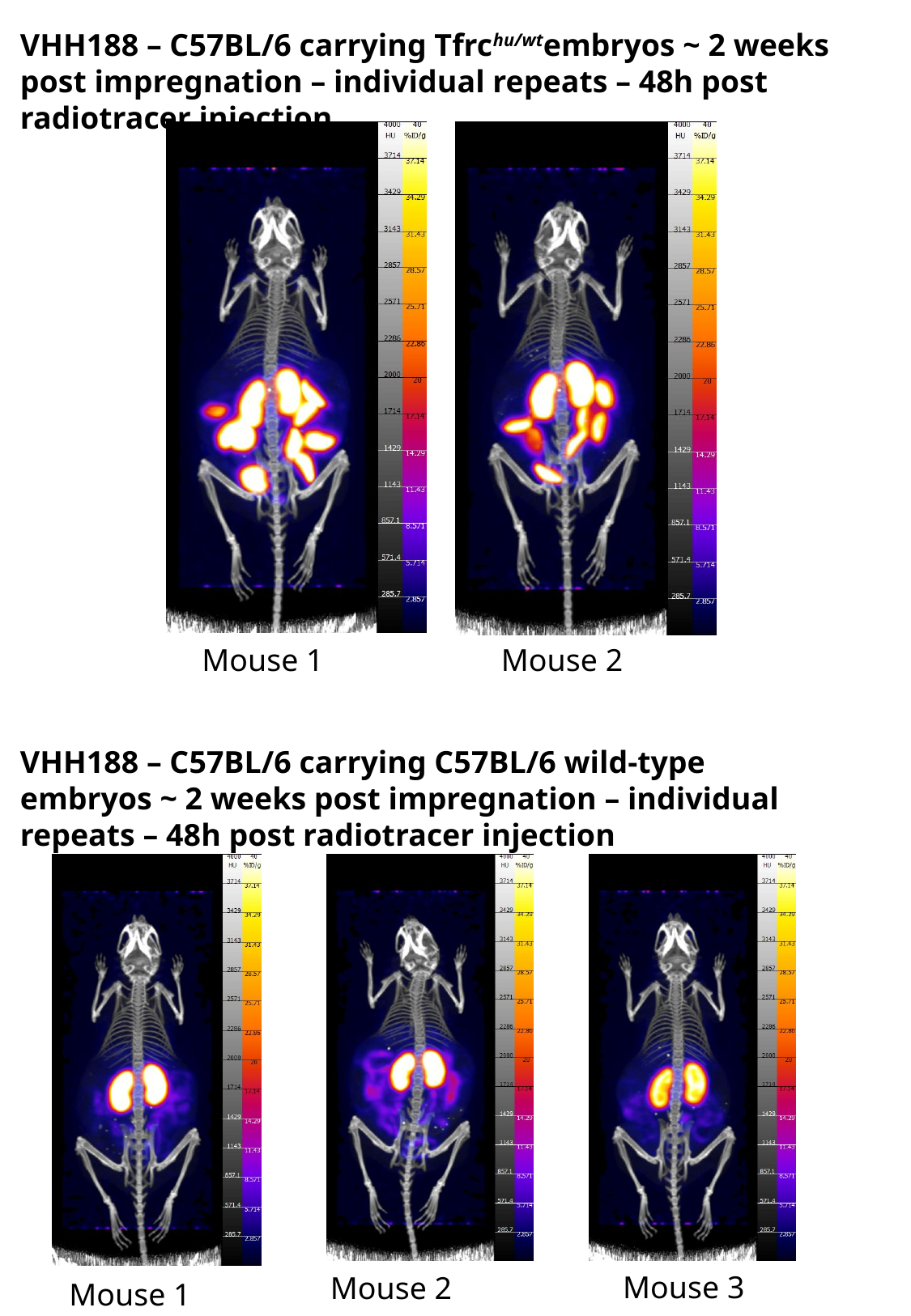

VHH188 – C57BL/6 carrying Tfrchu/wtembryos ~ 2 weeks post impregnation – individual repeats – 48h post radiotracer injection
Mouse 1
Mouse 2
VHH188 – C57BL/6 carrying C57BL/6 wild-type embryos ~ 2 weeks post impregnation – individual repeats – 48h post radiotracer injection
Mouse 3
Mouse 2
Mouse 1

## Slide 3
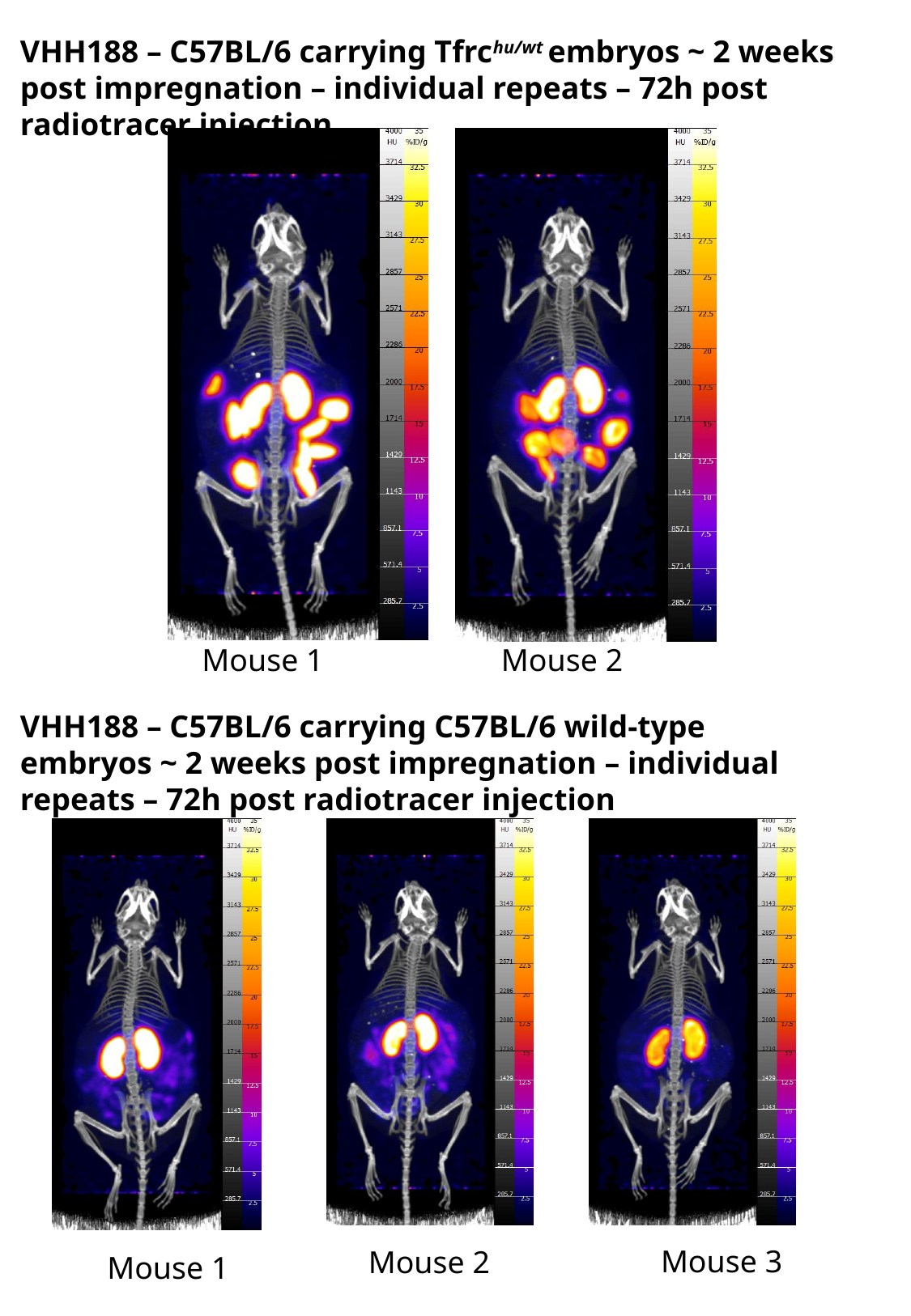

VHH188 – C57BL/6 carrying Tfrchu/wt embryos ~ 2 weeks post impregnation – individual repeats – 72h post radiotracer injection
Mouse 1
Mouse 2
VHH188 – C57BL/6 carrying C57BL/6 wild-type embryos ~ 2 weeks post impregnation – individual repeats – 72h post radiotracer injection
Mouse 3
Mouse 2
Mouse 1
